# Structure and biosynthesis of deoxy-polyamine in *Xenorhabdus bovienii*

**DOI:** 10.1093/jimb/kuab006

**Published:** 2021-01-25

**Authors:** Sebastian L Wenski, Natalie Berghaus, Nadine Keller, Helge B Bode

**Affiliations:** Molekulare Biotechnologie, Fachbereich Biowissenschaften, Goethe Universität Frankfurt, 60438 Frankfurt, Germany; Molekulare Biotechnologie, Fachbereich Biowissenschaften, Goethe Universität Frankfurt, 60438 Frankfurt, Germany; Molekulare Biotechnologie, Fachbereich Biowissenschaften, Goethe Universität Frankfurt, 60438 Frankfurt, Germany; Molekulare Biotechnologie, Fachbereich Biowissenschaften, Goethe Universität Frankfurt, 60438 Frankfurt, Germany; Buchmann Institute for Molecular Life Sciences (BMLS), Goethe Universität Frankfurt, 60438 Frankfurt, Germany; Senckenberg Gesellschaft für Naturforschung, 60325 Frankfurt, Germany; Department of Natural Products in Organismic Interactions, Max Planck Institute for Terrestrial Microbiology, 35043 Marburg, Germany

**Keywords:** Biological activity, Fabclavine, Natural products, Polyunsaturated fatty acid biosynthesis, PKS engineering

## Abstract

Polyamine moieties have been described as part of the fabclavine and zeamine family of natural products. While the corresponding biosynthetic gene clusters have been found in many different proteobacteria, a unique BGC was identified in the entomopathogenic bacterium *Xenorhabdus bovienii*. Mass spectrometric analysis of a *X. bovienii* mutant strain revealed a new deoxy-polyamine. The corresponding biosynthesis includes two additional reductive steps, initiated by an additional dehydratase (DH) domain, which was not found in any other *Xenorhabdus* strain. Moreover, this DH domain could be successfully integrated into homologous biosynthesis pathways, leading to the formation of other deoxy-polyamines. Additional heterologous production experiments revealed that the DH domain could act *in cis* as well as *in trans*.

## Introduction

There has been a constant interest in polyketide synthases (PKS) and natural products derived thereof since the identification of erythromycin, an antibiotic identified in the early 1950s (Demain, 2014; Robbins et al., 2016). In general, a minimal set of domains in these multidomain multifunctional giant enzymes includes a β-ketoacylsynthase (KS), an acyl transferase domain (AT) and an acyl carrier protein (ACP) (Hertweck, 2009). Following the Claisen condensation of the acyl units, the reduction of the β-keto group is optional and requires additional domains like a ketoreductase (KR), a dehydratase (DH) and/or an enoyl reductase (ER) domain (Hertweck, 2009; Nivina et al., 2019; Robbins et al., 2016). In contrast, biochemically closely related fatty acid synthases (FAS) reduce the β-keto group completely after each elongation of the acyl chain (Hertweck, 2009). A combination of both pathways can be observed for the biosynthesis of polyunsaturated fatty acids (PUFAs), which is described for marine proteobacteria as well as terrestrial myxobacteria and thraustochytrids (Gemperlein et al., 2014; Metz et al., 2001, 2009; Shulse & Allen, 2011). Natural products resulting from the PUFA biosynthesis gene cluster (*pfa* BGC) are the long chain PUFAs eicosapentaenoic acid (EPA, 20:5, *n*-3) or docosahexaenoic acid (DHA, 22:6, *n*-3). Both show anti-inflammatory properties which might help in human chronic diseases like diabetes or obesity (Lorente-Cebrián et al., 2013). Furthermore, the colonization of the gastric mucosa in mice by *Helicobacter pylori*, whose infection in humans is associated with several gastric diseases, can be reduced by EPA supplementation (Yamamoto et al., 2016).

**Fig. 1:**
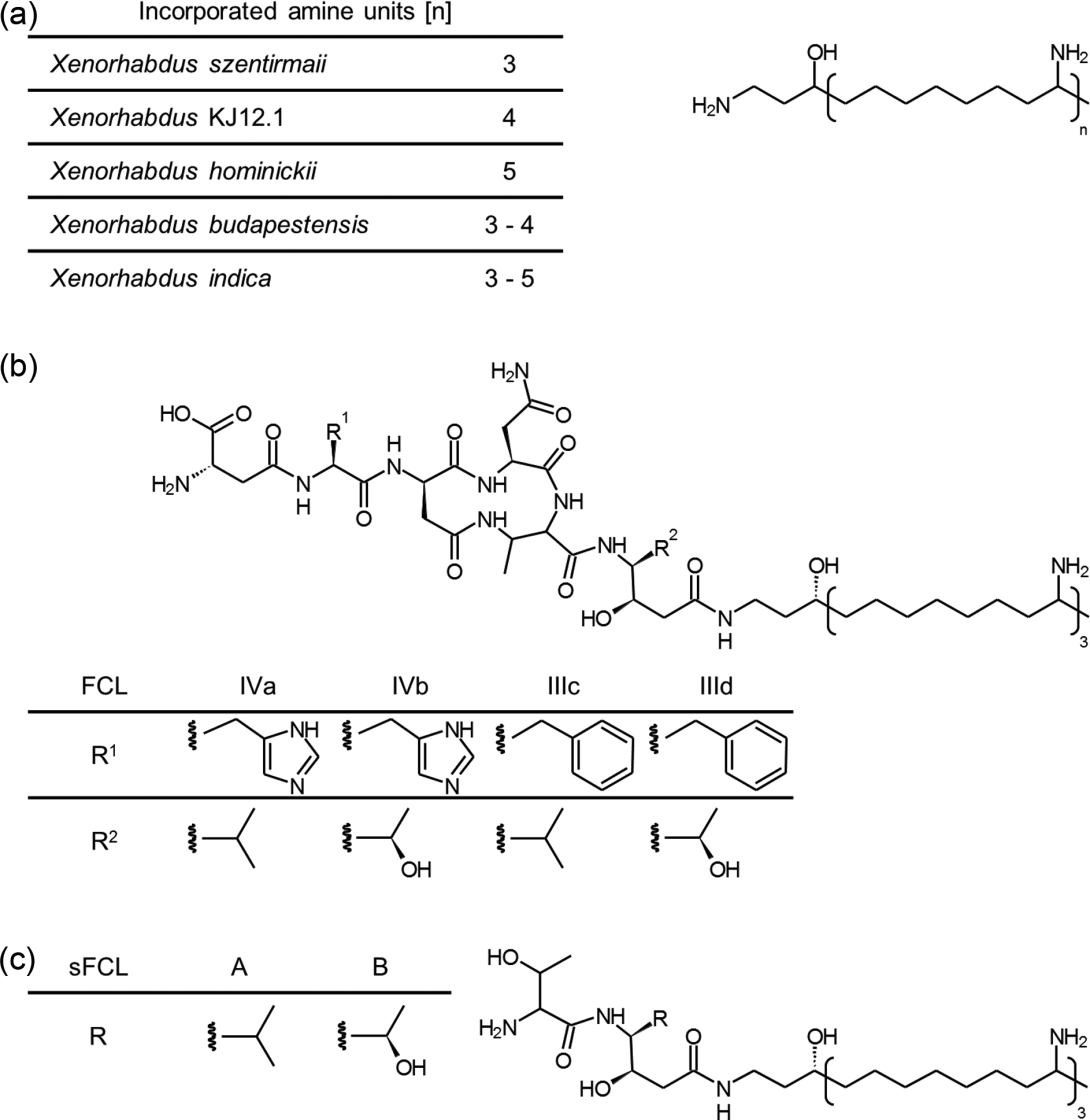
Types of fabclavines. Shown are polyamines from different *Xenorhabdus* strains (a) as well as examples of the full-length (FCL) (b) and shortened derivatives (sFCL) (c) from *X. szentirmaii* (Wenski et al., 2019, 2020).

Homologous *pfa* genes can be found in several microorganisms for which the production of PUFAs or derivatives thereof is mostly unknown (Shulse & Allen, 2011). Furthermore, a PUFA-related biosynthesis was observed in *Serratia plymuthica* and *Xenorhabdus budapestensis*, both producing the closely related zeamine and fabclavine natural products, respectively (Fuchs et al., 2014; Masschelein et al., 2015). Biochemically, both compound classes show a highly complex biosynthesis including nonribosomal peptide synthases (NRPS) and a type I PKS beside the PUFA-like biosynthesis (Fig. S1) (Fuchs et al., 2014; Masschelein et al., 2015; Wenski et al., 2019). Resulting products in *Xenorhabdus* strains are full-length (FCL) as well as shortened fabclavines (sFCL), named here as sFcl-A and sFcl-B, and polyamines (Fig. 1) (Wenski et al., 2019). While full-length derivatives show a broad-spectrum bioactivity, the roles of the shortened fabclavines and the polyamine part are not fully understood yet (Fuchs et al., 2014; Wenski et al., 2019).

Fabclavine-producing BGCs (*fcl*) are widespread in *Xenorhabdus* strains and detailed analysis revealed a large chemical diversity of derivatives in each strain (Wenski et al., 2020). This diversity results from flexible adenylation domains, an iterative type I PKS as well as the PUFA-related biosynthesis part (Wenski et al., 2020). The type I PKS FclC and FclD as well as the ER domain from the bi-functional enzyme FclE are related to PUFA-biosynthesis, which are extended by the aminotransferase domain of FclE, the 3-oxoacyl-ACP reductase FclF and the thioester reductase FclG (Fuchs et al., 2014; Wenski et al., 2019). Strikingly, the resulting product is a long acyl chain, substituted with amine moieties instead of unsaturated double bonds, as they would be expected for a PUFA-like biosynthesis product (Fig. 1). The polyamine is generated in multiple cycles by elongation with malonate units, with the intermediary β-keto moiety being either transaminated to an amine or reduced (Fig. S1) (Wenski et al., 2019). Depending on the number of cycles, the resulting polyamine chain length differs from three to five eight-carbon amine units in different *Xenorhabdus* strains (Fig. 1) (Wenski et al., 2020).

In this work, the (fabclavine-like) polyamine biosynthesis in *Xenorhabdus bovienii* was elucidated, leading to the identification of a yet unknown deoxy-polyamine derived from an additional PKS-like DH domain as part of FclC.

## Results and Discussion

### *X. bovienii* Produces a Deoxy-Polyamine

**Fig. 2:**
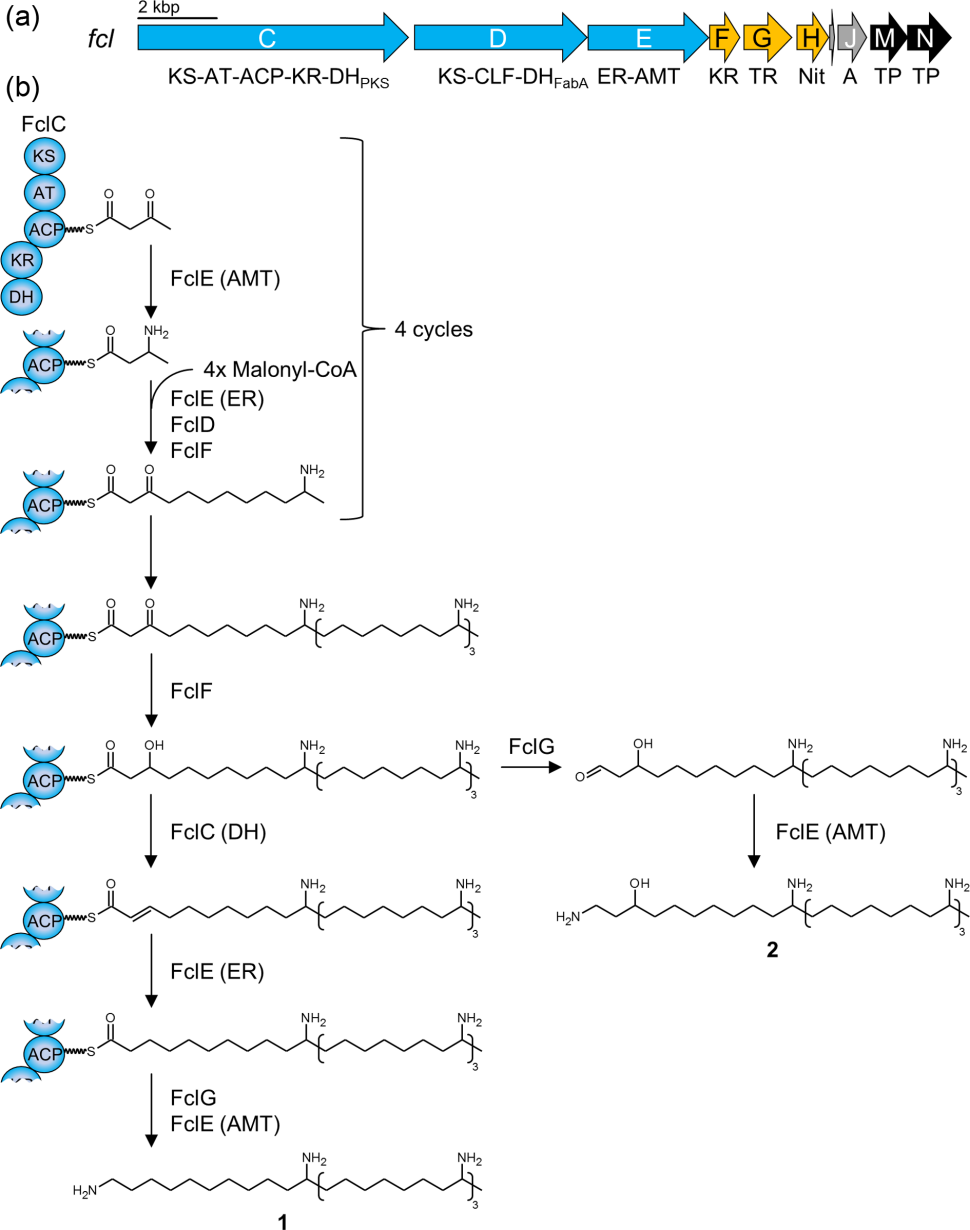
Fabclavine BGC with protein functions (a) and postulated biosynthesis of the polyamines in *X. bovienii* (b) (Wenski et al., 2019, 2020). The general pathway is based on the previously described biosynthesis in *X. szentirmaii* for the formation of **2** and is extended by two reductive steps for the formation of **1** (Wenski et al., 2019). Abbreviations: KS: ketosynthase, AT: acyltransferase, ACP: acyl carrier protein, KR: ketoreductase, DH: dehydratase (PKS- or FabA-like), CLF: chain length factor, ER: enoyl reductase, AMT: aminotransferase, TR: thioester reductase, Nit: nitrilase, A: adenylation, TP: transport.

Previous analysis of multiple *Xenorhabdus* strains revealed a wide distribution of the *fcl* BGC, resulting in a large structural diversity (Wenski et al., 2020). However, only genes responsible for polyamine formation and transport are encoded in *X. bovienii*, while the NRPS and PKS encoding genes usually present in *fcl* BGCs are not found or are truncated (Fig. 2) (Wenski et al., 2020). Since this suggests that *X. bovienii* is neither able to produce full-length nor shortened fabclavines but a polyamine-like compound, a promoter-exchange mutant was generated to identify corresponding products of this unusual *fcl* BGC (Wenski et al., 2020). In such mutants, promoter induction mostly leads to an overproduction of the activated BGC while without induction a ‘knock-out’ phenotype similar to a insertion into or to a deletion of the gene of interest is observed (Bode et al., 2015, 2019). Matrix-assisted laser desorption/ionization high resolution mass spectrometry (MALDI-HRMS) revealed two signals in the induced mutant with an observed *m/z* of 582.6383 [M + H]^+^ (**1**) and 598.6334 [M + H]^+^ (**2**) (Fig. S2). Compound **2** was identified as the already described four amine unit polyamine (C_36_H_79_N_5_O, Δppm −3.84) from *X. budapestensis* (Fuchs et al., 2014). Isotope-labeling experiments were performed to determine the exact number of incorporated carbon and nitrogen atoms in compound **1**, leading to the sum formula C_36_H_79_N_5_ that was confirmed via its high resolution mass with a Δppm of −4.29 (Figs S2 and S3). Therefore, comparison of the sum formulas of **1** and **2** suggested that **1** is the deoxy derivate of **2** (Fig. 2).

### An Additional Dehydratase Domain in FclC is Responsible for the Production of Deoxy-Polyamine

**Fig. 3:**
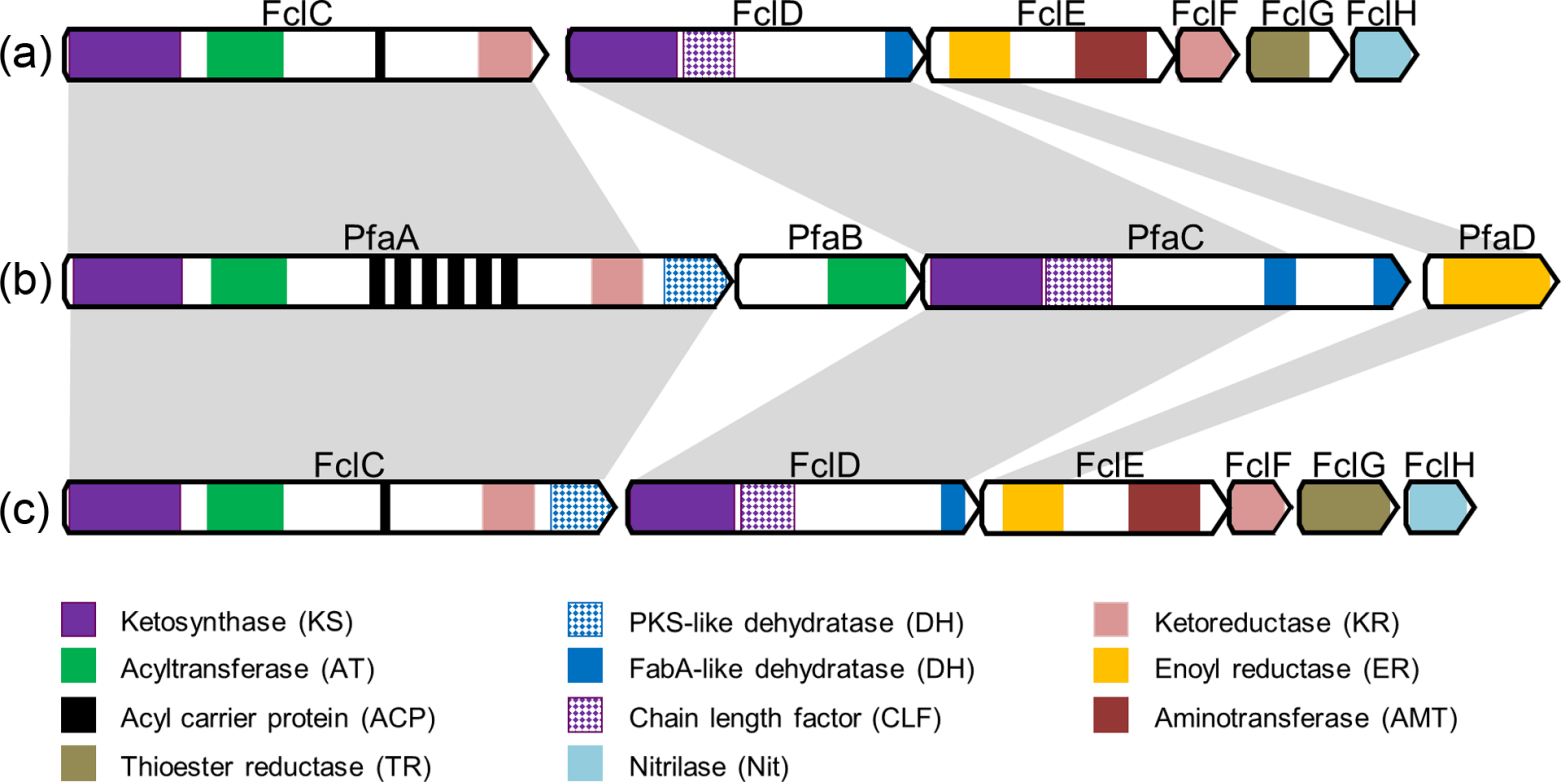
Domain organization of the fabclavine polyamine biosynthesis in *X. szentirmaii* DSM 16338 (a) and *X. bovienii* SS-2004 (c) in comparison with the EPA biosynthesis in *S. pneumatophori* strain SCRC-2738 (b). The domain organization of FclC from *X. szentirmaii* represents the major type of fabclavine producing *Xenorhabdus* strains (Fig. S4) (Wenski et al., 2020).

In general, fabclavine-producing strains like *X. szentirmaii* usually harbor only one FabA-like DH domain in FclD (Fig. 3 and Figs S4 and S5) (Wenski et al., 2020). However, detailed *in silico* analysis revealed a second DH domain in the C-terminus of FclC from *X. bovienii*, which is PKS-related (Fig. 3) (Wenski et al., 2020). Two different types of DH domains can also be observed in the PUFA biosynthesis of *Shewanella pneumatophori* (Fig. 3) (Gemperlein et al., 2014). Further homologues of the PKS-like DH domain from *X. bovienii* could be identified in multiple strains like the zeamine producing bacteria *S. plymuthica* and *Dickeya zeae, Photorhabdus temperata, Fischerella thermalis*, or *Agrobacterium tumefaciens* (Table S3) (Hellberg et al., 2015; Masschelein et al., 2015; Wu et al., 2010). However, the occurrence in the genus *Xenorhabdus* seems to be restricted to *X. bovienii* and its subspecies (Table S3 and Fig. S4).

**Fig. 4:**
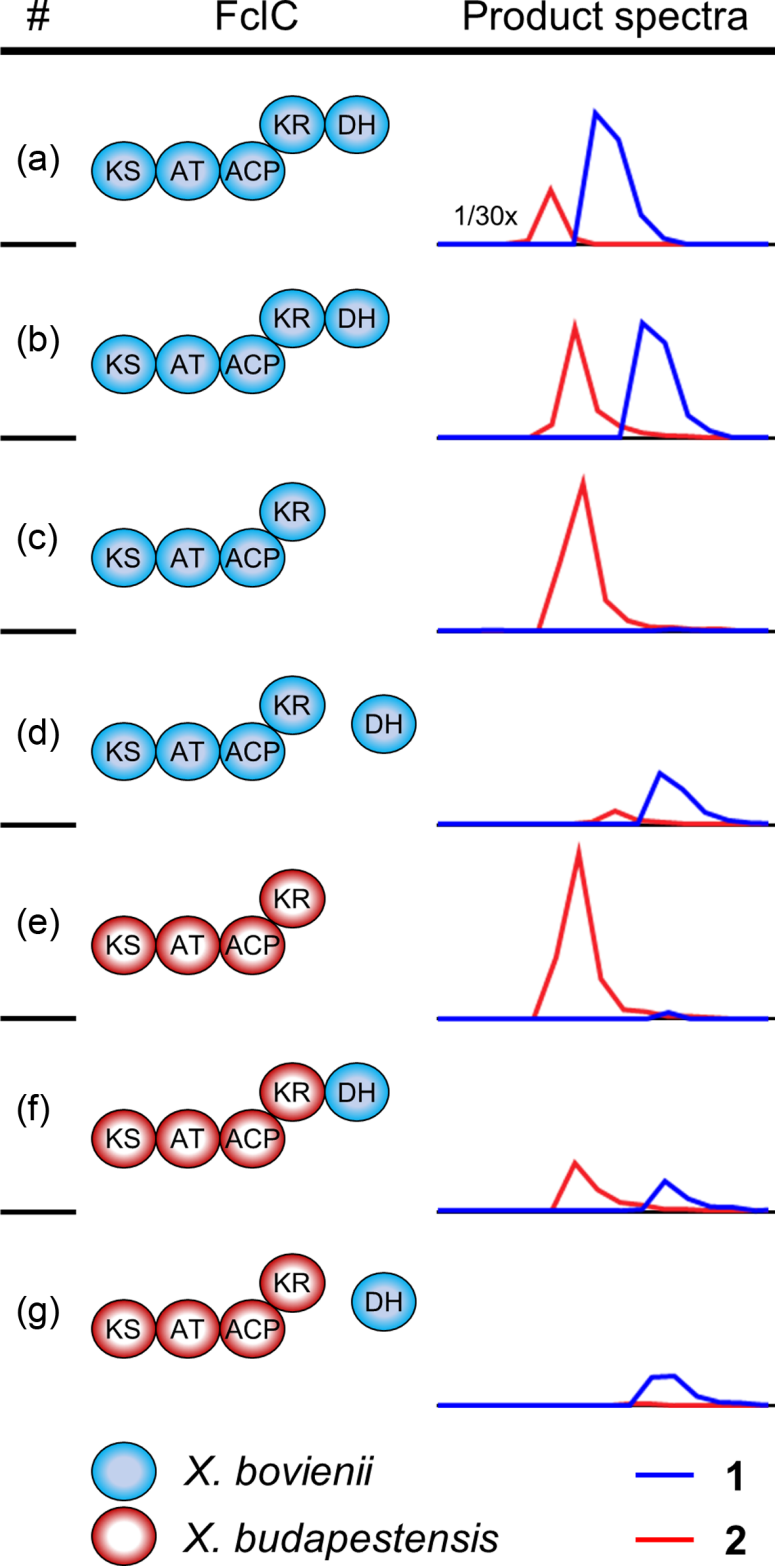
Product spectra of native and engineered FclC in the polyamine biosynthesis of *X. bovienii* and *X. budapestensis*. FclC was expressed together with FclDEFGH either in the native producer *X. bovienii* (a) or heterologously in *E. coli* (b–g) (Fig. 2). Domain organization and origin of FclC is shown as well as the extracted ion chromatograms of the double charged masses for **1** ([M + 2H]^2+^ 291.8241) and **2** ([M + 2H]^2+^ 299.8215) (Figs S8 and S9). Production titer in *X. bovienii* were 30× higher compared to that in *E. coli*. Abbreviations: KS: ketosynthase, AT: acyltransferase, ACP: acyl carrier protein, KR: ketoreductase, DH: dehydratase.

During the fabclavine biosynthesis in *X. szentirmaii* the genes *fclCDEFG* are essential for polyamine formation (Wenski et al., 2019). Furthermore, deletion of *fclH* leads to over 70 per cent decrease in the polyamine production titer (Fig. S6). Thus, *fclCDEFGH* from *X. bovienii* were cloned in an inducible plasmid for the heterologous production in *Escherichia coli*. Consequently, induced and noninduced production cultures were analyzed by high-performance liquid chromatography high-resolution mass spectrometry (HPLC-HRMS). The production of the compounds **1** and **2** were confirmed in the promoter exchange mutant of *X. bovienii* as well as in *E. coli* (Fig. 4a and b). However, a comparison of the concentrations of **1** and **2** reveals a much lower production titer in *E. coli* (Table S5). To analyze the function of the additional PKS-like DH domain, its encoding region was removed from the *E. coli* plasmid (Fig. S7). Subsequent analysis revealed an increased production of **2** while the formation of **1** was abolished (Fig. 4c and Fig. S8). This confirmed that the additional DH domain is involved in the formation of the deoxy-polyamine. Based on these results the postulated biosynthesis is shown in Fig. 2: The recently described pathway leading to the formation of **2** is extended by two additional reduction steps (Wenski et al., 2019). The dehydration of the hydroxy group is optional and introduced by the PKS-like DH domain of FclC. The resulting enoyl-derivate is suggested to be further reduced by FclE, harboring the only ER domain encoded in the *fcl* BGC (Fig. 2). However, it cannot be excluded that another ER domain from the production strain is responsible for this catalytic step. Referring to the recently published biosynthesis, the resulting intermediate is reductively released and transaminated to form the final deoxy-polyamine (**1**) (Fig. 2) (Wenski et al., 2019).

Recently published studies showed that during the PUFA-biosynthesis in *Aureispira marina* the PKS-like DH domain is required for the dehydration in the early stages of arachidonic acid formation and an inactivation leads to dramatic decrease in the production titer (Hayashi et al., 2019). However, during polyamine biosynthesis the loss of the PKS-like DH domain led to the exclusive production of a hydroxylated polyamine as it can be observed for the deletion mutant or in further fabclavine producing strains (Figs 1 and 4) (Wenski et al., 2020). This indicates that this domain is only required in the late stages of the biosynthesis, while all other dehydration steps during chain elongation and saturation can be performed by the FabA-like DH domain of FclD.

### Addition of DH Domain Changes Products to Deoxy-Polyamines

The manipulation of reductive loops (KR, DH and ER domain) is an effective tool for PKS engineering to change the product spectrum (Bozhüyük et al., 2019; Keatinge-Clay, 2012; Klaus & Grininger, 2018; Weissman, 2016). Due to its nonessential role during the chain elongation, the PKS-like DH domain of *X. bovienii* might be used in engineering approaches. Previously published studies showed that FclC is highly conserved among the different fabclavine producing strains except for the occurrence of the DH domain in *X. bovienii* (Wenski et al., 2019; Wenski et al., 2020). Detailed alignments revealed that multiple strains including *X. bovienii, X. budapestensis,* and *X. hominickii* share a common motif C-terminal of the KR domain (Fig. S5). This so-called ‘YxAxK’-motif was identified as last conserved part before the protein identity in FclC decreases significantly. In an engineering approach, this motif was used as junction to covalently fuse the PKS-like DH domain from *X. bovienii* to FclC from *X. budapestensis* (Fig. S7), which originally produces polyamine **2** (Fig. 4e and Fig. S9). Subsequent HPLC-analysis showed that the engineered FclC is functional, resulting in the product formation of **1** as well as **2** (Fig. 4f and Fig. S9). Comparable results were observed during the manipulation of polyamine biosynthesis in *X. hominickii*, which naturally produces a five-amine unit polyamine (Fig. 1 and Figs S10 and S11) (Wenski et al., 2020). In summary, these results highlight that the DH domain initiates the formation of the deoxy-polyamine, and further showed its compatibility with homologous FclC enzymes from other fabclavine producing strains.

### The DH Domain can also Act *in trans*

After the successful covalent fusion of the DH domain to homologous FclC enzymes, further experiments analyzed the ability of this domain to act *in trans* as stand-alone domain. Therefore, *E. coli* strains were used encoding *fclCDEFGH* from *X. budapestensis* or *fclC*(Δ*DH*)*DEFGH* from *X. bovienii*. Both *E. coli* strains produce compound **2** (Fig. 4c and e). Furthermore, the additional DH domain from *X. bovienii* (exact amino acid sequence is shown in Fig. S7) was cloned into a plasmid and coexpressed in both *E. coli* strains. Subsequent HPLC analysis of the mutants revealed that the plasmid-based coexpression of the stand-alone DH domain changed the product formation to **1**, while **2** was not produced at all or at much lower levels (Fig. 4d and g). For the polyamine biosynthesis of *X. hominickii* a similar shift from the hydroxylated to the deoxy derivate was observed (Figs S10 and S11). This confirmed the ability of the DH domain to act *in trans*. A recently published study showed comparable results: PKS- and FabA-like DH domains from the PUFA-biosynthesis of *Thraustochytrium* were expressed as stand-alone enzymes in an *E. coli* mutant and were able to restore the defective phenotype (Xie et al., 2018).

### Deoxy-Polyamine can be Incorporated into Full-Length Fabclavine

In the fabclavine biosynthesis, the polyamine is connected with the enzyme-bound NRPS-PKS-intermediate by the condensation-domain like protein FclL, which showed relaxed substrate specificity with respect to polyamine chain length (Fig. S1) (Wenski et al., 2019). Consequently, the four-amine unit polyamine from *X. budapestensis* as well as the five-amine unit polyamine from *X. hominickii* can be integrated into the biosynthesis of *X. szentirmaii* (Fig. 1 and Fig. S12) (Wenski et al., 2019, 2020). Nevertheless, small amines like pentylamine or spermine were not accepted (Wenski et al., 2019). Hence, we were interested in the role of the hydroxy group for polyamine recognition by FclL. As the deoxy-polyamine from *X. bovienii* lacks this chemical moiety, the conjugation with the NRPS-PKS-intermediate from *X. szentirmaii* was analyzed. Therefore, the polyamine-deficient mutant *X. szentirmaii* ∆*fclCDE* was complemented with the polyamine-forming genes *fclCDEFGH* from *X. bovienii*. MALDI-MS analysis of the induced production culture revealed a signal with a *m/z* of 1290.93 [M + H]^+^ (C_64_H_119_N_15_O_12_), corresponding to a fabclavine hybrid, which consists of a NRPS-PKS-part from *X. szentirmaii* and the deoxy-polyamine from *X. bovienii* (Fig. S12). These results confirmed that the hydroxy group of the polyamine is not essential for the FclL-catalyzed condensation with the NRPS-PKS-part.

### Polyamines are the Smallest Bioactive Fabclavine Parts

The elucidation of the fabclavine biosynthesis revealed that *X. szentirmaii* produces full-length and shortened fabclavines as well as the polyamine (Fig. 1) (Wenski et al., 2019). However, only for the full-length products a bioactivity was confirmed previously (Fuchs et al., 2014; Wenski et al., 2020). Therefore, mutants with deletions of *fclK* or *fclI* were generated, leading to a modified fabclavine biosynthesis with an exclusive production of the polyamine alone or together with the parallel production of the shortened derivatives (Bode et al., 2015, 2019; Wenski et al., 2019). Subsequent analyses revealed bioactivity of the shortened fabclavines and the polyamine from *X. szentirmaii* against selected microbial strains (Table S4). Finally, we were interested in the polyamines of *X. hominickii, Xenorhabdus* KJ12.1 or *X. bovienii*, which differ in the number of incorporated amine units or the hydroxy group (Figs 1 and 2) (Wenski et al., 2020). While culture supernatants without known polyamine titer were used, preventing a quantitative comparison between the different polyamines, qualitative analyses confirmed their bioactivity (Table S4).

## Conclusion

In this work, the (fabclavine) polyamine biosynthesis in *X. bovienii* was elucidated, revealing a novel deoxy-polyamine (**1**) beside an already described polyamine **2** (Fig. 2). The corresponding *fcl* BGC encodes an additional PKS-like DH domain in FclC, which occurs exclusively in *X. bovienii* within the genus *Xenorhabdus* (Table S3 and Fig. S4). This additional domain initiates a dehydration step at the β-hydroxy group of the full length intermediate, followed by an enoyl reduction, leading to the formation of **1**. Furthermore, this PKS-like DH domain was successfully introduced into the homologous (fabclavine) polyamine biosynthesis of *X. budapestensis* and *X. hominickii*, both possessing naturally only the FabA-like DH domain in FclD. Thereby, deoxy-polyamines were produced independently of the PKS-like DH domain being covalently fused to FclC or coexpressed as stand-alone DH domain.

Following the previously identified large diversity of fabclavine derivatives among the genus *Xenorhabdus*, this additional DH domain seems to be another diversification mechanism during fabclavine biosynthesis (Wenski et al., 2020). Future work will show if this DH domain can also be used to manipulate nonfabclavine like PKS biosynthesis pathways.

## Supplementary Material

kuab006_Supplemental_File

## Data Availability

All data used for this manuscript is clearly made available in form of citing previous publications or giving the detaily in the supporting information.
