# Supplementary material for: Structure and biosynthesis of deoxy-polyamine in Xenorhabdus bovienii
Source: J Ind Microbiol Biotechnol. 2021 Jan 25;48(3-4):kuab006. doi: 10.1093/jimb/kuab006 (PMC9113149; doi:10.1093/jimb/kuab006)
Supplement: kuab006_Supplemental_File [file kuab006_Supplemental_File.docx]

**Structure and biosynthesis of deoxy-polyamine in *X. bovienii***

Sebastian L. Wenski^1^, Natalie Berghaus^1^, Nadine Keller^1^, Helge B. Bode^1,2,3,4,^*

^1^ Molekulare Biotechnologie, Fachbereich Biowissenschaften, Goethe Universität Frankfurt, 60438 Frankfurt, Germany. E‑mail: h.bode@bio.uni-frankfurt.de

^2^ Buchmann Institute for Molecular Life Sciences (BMLS), Goethe Universität Frankfurt, 60438 Frankfurt, Germany.

^3^ Senckenberg Gesellschaft für Naturforschung, 60325 Frankfurt, Germany

^4^ Max Planck Institute for Terrestrial Microbiology, Department of Natural Products in Organismic Interactions, 35043 Marburg, Germany

**Material and methods**

1. **Strain cultivation**

The strains were cultivated as described previously [15]: Briefly, *Xenorhabdus* or *E. coli* strains were cultivated on LB-agar plates or in liquid LB media at 30°C or 37°C. In general, LB-media was inoculated with an overnight grown pre-culture, production induced with 0.2 % L-arabinose and cultivated with constant shaking for 1-3 days at 30°C. For the HPLC-HRMS measurements of the polyamine production in *E. coli* and *X. bovienii* XPPM-media was used and after inoculation the cultures were incubated for 3 h at 30°C before 0.2% L-arabinose was added [2]. Kanamycin [50 μg/ml], chloramphenicol [34 μg/ml] or aminolevulinic acid (ALA) [50 μg/ml] were added if required.

1. Isotope-labeling experiments

Overnight grown cultures were washed three times with H_2_O and the cells were used for inoculation of ISOGRO®-13C or ISOGRO®-15N growth media (Sigma-Aldrich), supplemented with Kanamycin [50 μg/ml] and 0.2% L-arabinose if required. Cultures were incubated with shaking at 30°C for 2 d.

1. **Bioactivity assay**

For the bioactivity assay the corresponding strains were inoculated with an OD_600_ of 0.1 without antibiotics, if appropriate induced with 0.2% L-arabinose and cultivated for 72 h with constant shaking (200 rpm) at 30°C. After centrifugation, the culture supernatant was heated for 10-15 min at 95°C and again centrifuged for 15 min and subsequent used as processed sample. Overnight cultures of the tested microbes were adjusted to a specific OD_600_ (*C. lusitaniae/ E. coli*: 0.5; *S. cerevisiae*: 1) and subsequent coated on agar-plates. Wells with a 12 mm diameter were perforated into the agar and filled with 200 µl processed sample (n=3), followed by cultivation for 2 d at 30°C. The inhibitory activity was determined as the diameter of the inhibition zone deducting the diameter of the well in mm.

1. **Generation of deletion and promoter exchange mutants**

The generation of deletion or promoter exchange mutants was performed as described previously and corresponding oligonucleotides can be found in Table S2 [4, 15]: Promoter exchange vectors were generated via amplification of 300-1000 bp of the start of the corresponding gene and cloning it into the either PCR-amplified or digested vector pCEP_kan [1]. Deletion vectors were generated via amplification of approximately 1000 bp of the up- and downstream region of the corresponding gene and cloning it into the either PCR-amplified or digested vector pEB17 [2, 9]. *E. coli* S17 or ST18 were transformed with corresponding vectors and conjugated with the *Xenorhabdus* strain as described previously [9, 11, 12].

1. **Heterologous polyamine production and addition/deletion of dehydratase domain**

The cloning of the heterologous polyamine producing plasmids of *X. bovienii* and *X. hominickii* was performed as described previously and corresponding oligonucleotides can be found in Table S2 [15]: The polyamine responsible biosynthesis genes *fclCDEFGH* were amplified from the genome of the corresponding strain in 3 parts with overlaps for extension PCR and yeast homologous recombination (ExRec) [10]. For each construct the corresponding fragments and the EcoRI/SgsI digested plasmid pFF1 were transformed into yeast by the protocols by Gietz and Schiestl [3, 7, 8]. After isolation the plasmids were re-transformed into *E. coli* DH10B::*mtaA*. For the exchange of the polyamine in *X. szentirmaii*, its corresponding ∆*fclCDE*-mutant strain was transformed with the heterologous polyamine producing plasmids according to [15].

For the covalently addition or deletion of the dehydratase domain from *X. bovienii* (amino acid sequences are shown in Figure S7) the confirmed pFF1_*fclCDEFGH* vectors were restricted with two different enzymes to linearize the plasmids (*X. bovienii*: SgrAI/SwaI; *X. budapestensis*: BcuI/SmiI; *X. hominickii*: AleI-v2/SwaI). For the PKS-like DH domain deletion in the *X. bovienii* construct (pFF1_*fclC*(∆DH)*DEFGH*) the flanking regions of the DH domain were amplified from *X. bovienii* and cloned into the linearized pFF1_*fclCDEFGH* vector by a Hot fusion assembly and transformed into *E. coli* DH10B::*mtaA* [5]. For the PKS-like DH domain addition constructs (pFF1_*fclC*(+DH)*DEFGH*) the flanking regions were amplified from the genome of *X. budapestensis* or *X. hominickii* and a third fragment, coding for the DH domain from *X. bovienii*, were cloned in between (direct after the YxAxK-motif). The three fragments were combined by Fusion PCR, cloned into the linearized pFF1_*fclCDEFGH* vector by a Hot fusion assembly and transformed into *E. coli* DH10B::*mtaA* [5]. The corresponding oligonucleotides for the deletion or addition of the DH domain can be found in Table S2.

1. **Cloning of dehydratase domain from FclC *X. bovienii* and co-expression**

For the co-production experiments we generated an inducible plasmid containing the separated PKS-like DH domain from *X. bovienii*. Therefore, the coding region of the DH domain (Start with an integrated methionine after the YxAxK-motif until the end of XBJ1_2953; amino acid sequences are shown in Figure S7) was amplified and cloned into the PCR-amplified vector plasmid pACYC_ara_tacI by Hot fusion assembly [5, 14]. The plasmid pACYC_ara_tacI_DH *X. bovienii* was transformed into electrocompetent *E. coli* DH10B::*mtaA*, which were already containing the pFF1_*fclCDEFGH* plasmid for the polyamine biosynthesis.

1. **Matrix-assisted Laser Desorption/Ionization Mass Spectrometry**

MALDI-MS measurements were performed as described previously [15, 16] : Briefly, 0.3 µl of a production culture (in LB-media) were spotted on a MALDI target and mixed with 0.25 µl 1:10 diluted ProteoMass Normal Mass Calibration Mix [ProteoMass™ MALDI Calibration Kit, Sigma-Aldrich] for internal calibration (only for high resolution measurements) and 0.9 µl α-cyano-4-hydroxycinnamic acid (CHCA) matrix [3 mg/ml in 75% acetonitrile, 0.1% trifluoroacetic acid]. The dried sample spot was coated with 5% formic acid in water. After the removal of the 5% formic acid solution the sample spot was mixed again with 0.6 µl CHCA. Cell MALDI measurements were performed with a MALDI LTQ Orbitrap XL [Thermo Fisher Scientific, Inc., Waltham, MA] instrument with a nitrogen laser at 337 nm in FTMS scan mode either with 60.000 or high resolution. Data analysis was performed with Qual Browser version 2.0.7 [Thermo Fisher Scientific].

1. **High Resolution High Performance Liquid Chromatography Mass Spectrometry**

For HPLC-HRMS measurements the heterologous production cultures (in XPPM-media) were incubated with equal volume of 2% formic acid in MeOH for 1 h at 30°C. The *X. szentirmaii* cultures (in LB-media) were incubated with equal volume MeOH. If required production cultures were previously fivefold concentrated via lyophilization due to low signal intensities. Subsequent to centrifugation the supernatant was transferred and utilized as sample. A volume of 30 µl (20 µl for *X. szentirmaii* cultures) was injected into a Dionex Ultimate 3000 HPLC coupled to a Bruker Impact^TM^ II ESI-Q-OTOF instrument set to positive ionization mode. Separation was performed on a C18 [Waters, ACQUITY UPLC BEH, 50mm x 2.1 mm x 1.7 μm] column with acetonitrile [0.1% formic acid] in H_2_O [0.1% formic acid] as solvent. The flow rate was 0.4 ml/min with the following gradient: 0-2 min 5 % ACN, 2-14 min 5-95 % ACN, 14-15 95 % ACN, 15-16 min 5 % ACN [3, 13]. The MS settings are described in detail in [13]. However, a mass range of m/z 100-800 was used. Data analysis was performed with the software DataAnalysis 4.3 [Bruker].

1. **Quantification of polyamine production titers**

For the quantification of **1** and **2** the polyamine spermine was used as standard. Therefore, different spermine concentrations (1000, 100, 10, 1, 0.1 µg/mL in H_2_O) were measured via HPLC-HRMS in triplicates. Using the peak area of each concentration a calibration curve was generated and utilized for the calculation of the production titers of **1** and **2**.

**Supplementary Tables**

**Table S1.** Strains used in this work. *E. coli*: *Escherichia coli*, *S. cerevisiae*: *Saccharomyces cerevisiae*, *C. lusitaniae*: *Candida lusitaniae*, *X. szentirmaii*: *Xenorhabdus szentirmaii*, *Xenorhabdus* KJ12.1, *X. hominickii*: *Xenorhabdus hominickii*, *X. bovienii*: *Xenorhabdus bovienii*, DH: dehydratase domain, Fcl: full-length fabclavine; sFcl: shortened fabclavine; PolyA: polyamine.

| **Strain** | **Application** | **Description** | **Origin** |
| --- | --- | --- | --- |
| *E. coli* S17 λ1-pir | Conjugation | - | [11] |
| *E. coli* ST18 | Conjugation | - | [12] |
| *S. cerevisiae* CEN.PK 2-1C | Bioactivity assay and yeast cloning | - | Euroscarf |
| *C. lusitaniae* DSM 70102 | Bioactivity assay | - | DSMZ |
| *E. coli* DH10B | Bioactivity assay | - | Invitrogen |
| *X. szentirmaii* DSM 16338 ∆*hfq* | Mutant in in ∆*hfq*-background | Deletion of *hfq* | [2] |
| *X. szentirmaii* DSM 16338 ∆*hfq* pCEP*_fcl* | Fcl/sFcl/PolyA-producer in ∆*hfq*-background | Deletion of *hfq*  Promoter exchange in front of *fclC* | [2] |
| *X. szentirmaii* DSM 16338 ∆*hfq* ∆*fclI* pCEP*_fcl* | sFcl/PolyA-producer  in ∆*hfq*-background | Deletion of *hfq* and *fclI*  Promoterexchange in front of *fclC* | This work |
| *X. szentirmaii* DSM 16338 ∆*hfq* ∆*fclK* pCEP*_fcl* | PolyA-producer  in ∆*hfq*-background | Deletion of *hfq* and *fclK*  Promoter exchange in front of *fclC* | This work |
| *Xenorhabdus* KJ12.1 ∆*hfq* ∆*fclK* pCEP*_fcl* | PolyA-producer  in ∆*hfq*-background | Deletion of *hfq* and *fclK*  Promoter exchange in front of *fclC* | This work |
| *X. hominickii* DSM 17903  ∆*hfq* ∆*fclK* pCEP*_fcl* | PolyA-producer  in ∆*hfq*-background | Deletion of *hfq* and *fclK*  Promoter exchange in front of *fclC* | This work |
| *X. bovienii* SS-2004  pCEP_*fcl* | PolyA-producer | Promoter exchange in front of *fclC* | This work |
| *E. coli* DH10B::*mtaA* | Yeast cloning and heterologous PolyA production |  | [10] |
| *E. coli* DH10B::*mtaA* pFF1*_fclCDEFGH X. bovienii* | Heterologous PolyA production | Heterologous production of *X. bovienii fclCDEFGH* | This work |
| *E. coli* DH10B::*mtaA* pFF1_*fclC*(∆DH)*DEFGH*  *X. bovienii* | Heterologous PolyA production | Heterologous production of *X. bovienii fclCDEFGH* with deleted DH domain in *fclC* | This work |
| *E. coli* DH10B::*mtaA* pFF1_*fclC*(∆DH)*DEFGH*  *X. bovienii*  pACYC_ara_tacI_DH  *X. bovienii* | Heterologous PolyA production | Heterologous production of *X. bovienii fclCDEFGH* with deleted DH domain in *fclC* and plasmid-based co-expression of the DH domain of *fclC* | This work |
| *E. coli* DH10B::*mtaA* pFF1*_fclCDEFGH*  *X. budapestensis* | Heterologous PolyA production | Heterologous production of *X.* *budapestensis fclCDEFGH* | [15] |
| *E. coli* DH10B::*mtaA* pFF1*_fclC*(+DH)*DEFGH*  *X. budapestensis* | Heterologous PolyA production | Heterologous production of *X.* *budapestensis fclCDEFGH* with additional DH domain of *fclC* from *X. bovienii* integrated in *fclC* *X. budapestensis* | This work |
| *E. coli* DH10B::*mtaA* pFF1*_fclCDEFGH*  *X. budapestensis*  pACYC_ara_tacI_DH  *X. bovienii* | Heterologous PolyA production | Heterologous production of *X.* *budapestensis fclCDEFGH* and plasmid-based co-expression of the DH domain of *fclC* from *X. bovienii* | This work |
| *E. coli* DH10B::*mtaA* pFF1_*fclCDEFGH*  *X. hominickii* | Heterologous PolyA production | Heterologous production of *X. hominickii fclCDEFGH* | This work |
| *E. coli* DH10B::*mtaA* pFF1_*fclC*(+DH)*DEFGH*  *X. hominickii* | Heterologous PolyA production | Heterologous production of *X. hominickii fclCDEFGH* with additional DH domain of *fclC* from *X. bovienii* integrated in *fclC* *X. hominickii* | This work |
| *E. coli* DH10B::*mtaA* pFF1_*fclCDEFGH*  *X. hominickii*  pACYC_ara_tacI_DH  *X. bovienii* | Heterologous PolyA production | Heterologous production of *X. hominickii fclCDEFGH* and plasmid-based co-expression of the DH domain of *fclC* from *X. bovienii* | This work |
| *X. szentirmaii* DSM 16338 ∆*fclCDE* | PolyA exchange in *X. szentirmaii* | Deletion of *fclCDE* in *X. szentirmaii* | [15] |

**Table S1 (continued).** Strains used in this work. *E. coli*: *Escherichia coli*, *S. cerevisiae*: *Saccharomyces cerevisiae*, *C. lusitaniae*: *Candida lusitaniae*, *X. szentirmaii*: *Xenorhabdus szentirmaii*, *Xenorhabdus* KJ12.1, *X. hominickii*: *Xenorhabdus hominickii*, *X. bovienii*: *Xenorhabdus bovienii*, DH: dehydratase domain, Fcl: full-length fabclavine; sFcl: shortened fabclavine; PolyA: polyamine.

| **Strain** | **Application** | **Description** | **Origin** |
| --- | --- | --- | --- |
| *X. szentirmaii* DSM 16338 ∆*fclCDE*  pFF1*_fclCDEFGH X. bovienii* | PolyA exchange in *X. szentirmaii* | Deletion of *fclCDE* in *X. szentirmaii* and heterologous production of *fclCDEFGH* from *X. bovienii* | This work |
| *X. szentirmaii* DSM 16338 ∆*fclCDE*  pFF1*_fclCDEFGH X. hominickii* | PolyA exchange in *X. szentirmaii* | Deletion of *fclCDE* in *X. szentirmaii* and heterologous production of *fclCDEFGH* from *X. hominickii* | This work |
| *X. szentirmaii* DSM 16338  ∆*fclK* | Influence of FclH | Deletion of *fclK* in *X. szentirmaii* | [15] |
| *X. szentirmaii* DSM 16338  ∆*fclK* ∆*fclH* | Influence of FclH | Deletion of *fclK* and *fclH* in *X. szentirmaii* | This work |

**Table S2.** Plasmids and oligonucleotides and their origin.

| **Plasmid** | **Locus tag(s) of analyzed gene(s)** | **Oligonucleotide** | **Sequence 5‘-3‘** |
| --- | --- | --- | --- |
| pCEP_*fcl*  *X. szentirmaii* | Xsze_03745 | [16] | [16] |
| ∆*fclI*  *X. szentirmaii* | Xsze_03739 | [15] | [15] |
| ∆*fclK*  *X. szentirmaii* | Xsze_03737 | [15] | [15] |
| ∆*fclH*  *X. szentirmaii* | Xsze_03740 | [15] | [15] |
| pCEP_*fcl*  *Xenorhabdus* KJ12.1 | Xekj_00388 | [16] | [16] |
| ∆*hfq*  *Xenorhabdus* KJ12.1 | Xekj_01457 | SW282_KJ12.1_LF_fw | CGATCCTCTAGAGTCGACCTGCAGCACTATCAATCGCTTTGCGTCAGC |
|  |  | SW283_KJ12.1_LF_rv | TGTCAGGCATTATCACTGATTCTATATTTTCCTTATTTTGTTGTTTTTAACTAAGAACCC |
|  |  | SW284_KJ12.1_RF_fw | CAAAATAAGGAAAATATAGAATCAGTGATAATGCCTGACATAAATAAAAACATAGG |
|  |  | SW285_KJ12.1_RF_rv | GAGAGCTCAGATCTACGCGTTTCATATGCTTCCAGAACACTGTCCACTGC |
| ∆*fclK*  *Xenorhabdus* KJ12.1 | Xekj_00380 | NB1_KJ_LF_fw | CGATCCTCTAGAGTCGACCTGCAGATCCTTGATAGCTTCCTCCATCC |
|  |  | NB2_KJ_LF_rv | TATCAGGTTGTCCGGGATCCGTTATTCCTCATTCCC |
|  |  | NB3_KJ_RF_ fw | GAGGAATAACGGATCCCGGACAACCTGATAGGC |
|  |  | NB4_KJ_RF_rv | GAGAGCTCAGATCTACGCGTTTCATATGGGCTGTCCAGATTGACAGGC |
| pCEP_*fcl*  *X. hominickii* | Xhom_02793 | [16] | [16] |
| ∆*hfq*  *X. hominickii* | Xhom_00479 | SW290_Xhom_LF_fw | CGATCCTCTAGAGTCGACCTGCAGCTGCATTGATTTATCGTGGAATGGATATCGG |
|  |  | SW291_Xhom_LF_rv | CGTCAGGTGTGGCCACTGATTCTATATTTTCCTTATTTTGTTGTTTTTAACTAAGAACCTATTGG |
|  |  | SW292_Xhom_RF_fw | CAAAATAAGGAAAATATAGAATCAGTGGCCACACCTGACG |
|  |  | SW293_Xhom_RF_rv | GAGAGCTCAGATCTACGCGTTTCATATGGGCAGCATAGACTTCAGCTGAGG |
| ∆*fclK*  *X. hominickii* | Xhom_02785 | SW329_Xhom_LF_fw | CGATCCTCTAGAGTCGACCTGCAGAAGGTACCAGTGTTCCCATCG |
|  |  | SW330_Xhom_LF_rv | ATGGTTCTCCTGCGGGATCTGTTATCCCTTTTCCCTTACC |
|  |  | SW331_Xhom_RF_fw | AAGGGATAACAGATCCCGCAGGAGAACCATGAAG |
|  |  | SW332_Xhom_RF_rv | GAGAGCTCAGATCTACGCGTTTCATATGCTCACTACCCGGTTGTTCCC |
| pCEP_*fcl*  *X. bov*ienii | XBJ1_2953 | SW151_Xbov_fw | TTTGGGCTAACAGGAGGCTAGCATATGTCTGAGACATATTTTTTGCATGATAGG |
|  |  | SW152_Xbov_rv | TCTGCAGAGCTCGAGCATGCACATCAACGCGACTTTCATCATAGACG |
| pFF1_*fclCDEFGH X. bovienii* | XBJ1_2948  -XBJ1_2953 | SW361_Xb_F1_fw | TTATCGCAACTCTCTACTGTTTCTCCATACCCGTTTTTTTGGGCTAACAGGAGGAATTCCATGTCTGAGACATATTTTTTGCATGATAGG |
|  |  | SW362_Xb_F1_rv | CGATTAATTATTTTCGCTATTCACCCTGTTCGATAGTTGACTGAGGAAAGATAATTTCCTCTATTCAACAAACACGGTAAATTTTGC |
|  |  | SW363_Xb_F2_fw | GAACAAGGCTATATTTATAGTCGGTTTCATAATGCAAAATTTACCGTGTTTGTTGAATAGAGGAAATTATCTTTCCTCAGTCAACTATCG |
|  |  | SW364_Xb_F2_rv | CGTTAGCGGTAACGCTGTCAGTTGATGGTTCGATTGATTCACAGATAAGCGCTGACTCATTTTAAAGCTTCCTGTTTCAGTTCTGC |
|  |  | SW365_Xb_F3_fw | ATACGAGCGACGCCAGCCACACCAGATCATGGCGGCAGAACTGAAACAGGAAGCTTTAAAATGAGTCAGCGCTTATCTGTG |
|  |  | SW366_Xb_F3_rv | CTTCACCTTTGCTCATGAACTCGCCAGAACCAGCAGCGGAGCCAGCGGATCCGGCGCGCCTTAGGTATCATTTCCTAACACATCG |
| pFF1_*fclC*(∆DH *)DEFGH*  *X. bovienii* | XBJ1_2948  -XBJ1_2953 | SW606 | GCACCCACCCATCGTACGTCATCGCCGGTGGTGGATTATTCTGCTCTGGCCAAAGTG |
|  |  | SW607 | AATTTCCTCTATTCAACAAACTCTCCGATCCCCAACGCC |
|  |  | SW608 | AGGCGTTGGGGATCGGAGAGTTTGTTGAATAGAGGAAATTATCTTTCCTCAG |
|  |  | SW609 | CAATGCCTGCTCAGCAGAATAAATAGTCCATTTAAATAAATTATCCAGCGAATTTAATTCTTCTTCC |
| pFF1_*fclCDEFGH*  *X. budapestensis* |  | [15] | [15] |
| pFF1_*fclC*(+DH)*DEFGH*  *X. budapestensis* | Xbud_02634-02639  +partial XBJ1_2953 | SW669 | GCCATGCAGGTCAGTCCCCATGATGTACTAGTTGTGACAGGGGGAGCCC |
|  |  | SW670 | CCCAACGCCTTAACTGTTTTCGGAGCCATATAAGTTTTCCCTCC |
|  |  | SW671 | CTTATATGGCTCCGAAAACAGTTAAGGCGTTGGGG |
|  |  | SW672 | CGTAATTTATTAGGTATCGTTTTCACGGTAAATTTTGCATTATGAAACC |
|  |  | SW673 | TTCATAATGCAAAATTTACCGTGAAAACGATACCTAATAAATTACGTAATGTAATC |
|  |  | SW674 | ATGCCTGATCTGCTGCATAGATTGTCCATTTAAATAGATCATCCAGTGTATTTAATTCTTCTTC |

**Table S2 (continued).** Plasmids and oligonucleotides and their origin.

| **Plasmid** | **Locus tag(s) of analyzed gene(s)** | **Oligonucleotide** | **Sequence 5‘-3‘** |
| --- | --- | --- | --- |
| pFF1_*fclCDEFGH*  *X. hominickii* | Xhom_02788  - Xhom_02793 | SW420_Xh_F1_fw | TTATCGCAACTCTCTACTGTTTCTCCATACCCGTTTTTTTGGGCTAACAGGAGGAATTCCATGTCTGAGTCATATCTTTTACATGATGG |
|  |  | SW421_Xh_F1_rv | TATTATTTATACCCGTTGTATTTCAAGTTGTCGCTTTATTTATTTCACTGCAACTGGATACAACC |
|  |  | SW422_Xh_F2_fw | ATCCAATAATATTCAGGTTGTATCCAGTTGCAGTGAAATAAATAAAGCGACAACTTGAAATACAACGG |
|  |  | SW423_Xh_F2_rv | GCCAATTGGCGGCCTGATTGATTCACCGATAAGCGCTGACTCATTTTGCTTCTTCCTGTTTCAGTTC |
|  |  | SW424_Xh_F3_fw | GGCAGGTGCGACAGAACTGAAACAGGAAGAAGCAAAATGAGTCAGCGCTTATCGGTGAATCAATC |
|  |  | SW425_Xh_F3_rv | CTTCACCTTTGCTCATGAACTCGCCAGAACCAGCAGCGGAGCCAGCGGATCCGGCGCGCCTTAATTCTTCAAAAGAAACGGATCTGCATC |
| pFF1_*fclC*(+DH)*DEFGH*  *X. hominickii* | Xhom_02788  - Xhom_02793  + partial XBJ1_2953 | SW652 | CAATCTGTGGTGGGATATTCTGCCACCAACGTGGCACCATCAGCACCGATGACG |
|  |  | SW653 | CCCAACGCCTTAACTGTTTTGGGCGCTTTATAAGTTTCTCCGC |
|  |  | SW654 | CTTATAAAGCGCCCAAAACAGTTAAGGCGTTGGGG |
|  |  | SW655 | TTTCTGCTGATATTAGTTTTCACGGTAAATTTTGCATTATGAAACC |
|  |  | SW656 | ATAATGCAAAATTTACCGTGAAAACTAATATCAGCAGAAATAATAACAGG |
|  |  | SW657 | GCCTGTTCAGAGGCGTAAATACTCCATTTAAATAAGTTATCCAGTAAGTTCAATTCTTCTTC |
| pACYC_ara_tacI (empty) | / | [14] | [14] |
| pACYC_ara_tacI*_*DH  *X. bovienii* | XBJ1_2953 | SW667 | CCATACCCGTTTTTTTGGGCTAACAGGAGGAATTCCATGACAGTTAAGGCGTTGGGG |
|  |  | SW668 | CGAGCCGATGATTAATTGTCAACAGCTCCTGCAGCTATTCAACAAACACGGTAAATTTTGC |

**Table S3.** Occurrence of homologous dehydratase domains in the genus *Xenorhabdus* and further strains. FclC homologs were identified by a BlastP search. Only one representative strain of a genus is shown. FclC from *X. szentirmaii* is shown as reference protein without DH domain, representing FclC-homologs from other *Xenorhabdus* strains (Fig. S4) [16].

|  | **Gene accession number** | **Protein size [Amino acids]** | **Identity [%]** |
| --- | --- | --- | --- |
| ***X. bovienii* subspecies** | | | |
| SS-2004 | CBJ82077.1 | 2274 | 100 |
| str. Jollieti | CDH27902.1 | 2274 | 100 |
| str. puntauvense | CDG98370.1 | 2277 | 94 |
| str. oregonense | CDH05851.1 | 2268 | 97 |
| str. kraussei Quebec | CDH18916.1 | 2289 | 88 |
| str. kraussei Becker Underwood | CDH23752.1 | 2290 | 94 |
| str. Intermedium | CDH34284.1 | 2309 | 69 |
| str. feltiae Moldova | CDH00683.1 | 2277 | 97 |
| str. feltiae France | CDG88351.1 | 2277 | 94 |
| str. feltiae Florida | CDG90959.1 | 2277 | 94 |
| **Further strains** | | | |
| *Fischerella thermalis* CCMEE5201 | PMB53690.1 | 2389 | 35 |
| *Dickeya zeae*  EC1 | AJC65778.1 | 2332 | 41 |
| *Photorhabdus temperata*  subsp. temperata Meg1 | KER04069.1 | 2218 | 49 |
| *Serratia plymuthica*  RVH1 | CCM44330.1 | 2248 | 49 |
| *Agrobacterium tumefaciens* | QCL88703.1 | 2373 | 37 |
| *Shewanella pneumatophori*  SCRC-2738 | AAB81123.1 | 2756 | 24.8 |
| **FclC-reference without DH domain** | | | |
| *X. szentirmaii* DSM 16338 | PHM32993.1 | 1995 | 72 |

**Table S4.** Inhibitory activity of the fabclavine types from *X. szentirmaii* and polyamines from different strains. Testorganisms were *Saccharomyces cerevisiae* CEN.PK2 (*S. cerevisiae*), *Candida lusitaniae* DSM 70102 (*C. lusitaniae*) and *Escherichia coli* DH10B (*E. coli*) against processed samples of induced (ind) and non-induced (non ind) mutant strains of *Xenorhabdus szentirmaii* DSM 16338 (*X. sze.*), *Xenorhabdus* KJ12.1 (KJ12.1), *X. hominickii* DSM 17903 (*X. hom*.) and *X. bovienii* SS-2004 (*X. bov*.). Structures corresponding to the strain-specific fabclavine types a, b and c are shown in Figure 1 and 2. Strains without deletion in the *fcl* BGC are able to produce full-length (b) and shortened fabclavines (c) and the polyamine (a), while a deletion of *fclI* shows only production of a and c, and a deletion in *fclK* shows only production of a [15]. The polyamine/fabclavine concentration in the different samples were neither determined nor normalized before the bioactivity assays.

| Analyzed strain | Fabclavine types | Inhibitory activity against tested microbe [mm] | | |
| --- | --- | --- | --- | --- |
|  |  | *S. cerevisiae* | *C. lusitaniae* | *E. coli* |
| *X. sze.* ∆*hfq*  pCEP_fcl (ind) | a, b, c | 18.3 | 21.7 | 12.7 |
| *X. sze.* ∆*hfq*  pCEP_fcl (non ind) | - | 0 | 0 | 0 |
| *X. sze.* ∆*hfq* ∆*fclI* pCEP_fcl (ind) | a, c | 4.3 | 3.7 | 0 |
| *X. sze.* ∆*hfq* ∆*fclI* pCEP_fcl (non ind) | - | 0 | 0 | 0 |
| *X. sze.* ∆*hfq* ∆*fclK* pCEP_fcl (ind) | a | 4 | 2.7 | 1 |
| *X. sze.* ∆*hfq* ∆*fclK* pCEP_fcl (non ind) | - | 0 | 0 | 0 |
| KJ12.1 Δ*hfq* Δ*fclK* pCEP_fclC (ind) | a (**2**) | 0 | 0 | 2 |
| KJ12.1 Δ*hfq* Δ*fclK* pCEP_fclC (non ind) | - | 0 | 0 | 0 |
| *X. hom.* ∆*hfq* ∆*fclK* pCEP_fcl (ind) | a | 0 | 1.3 | 2.7 |
| *X. hom.* ∆*hfq* ∆*fclK* pCEP_fcl (non ind) | - | 0 | 0 | 0 |
| *X. bov.*  pCEP_fclC (ind) | a (**1**, **2**) | 8.3 | 10.3 | 10.3 |
| *X. bov.*  pCEP_fclC (non ind) | - | 0 | 0 | 0 |

**Table S5.** Estimated production levels of compound **1** and **2** in *X. bovienii* SS-2004 pCEP_*fcl* and *E. coli* pFF1_*fclCDEFGH* *X. bovienii*. Since we were not able to isolate **1** and **2** using preparative chromatography, the structurally related polyamine spermine was used as standard allowing quantification of production cultures based on HPLC-HRMS. For that, different spermine concentrations (1000, 100, 10, 1, 0.1 µg/mL in H_2_O) were measured in triplicates to generate a calibration curve.

| Producing strain | Compound | Estimated production level |
| --- | --- | --- |
| *X. bovienii* pCEP_*fcl* | **1** | ~50 mg/L |
|  | **2** | ~10 mg/L |
| *E. coli* pFF1_*fclCDEFGH X. bovienii* | **1** | ~2 mg/L |
|  | **2** | ~1 mg/L |

**Supplementary Figures**


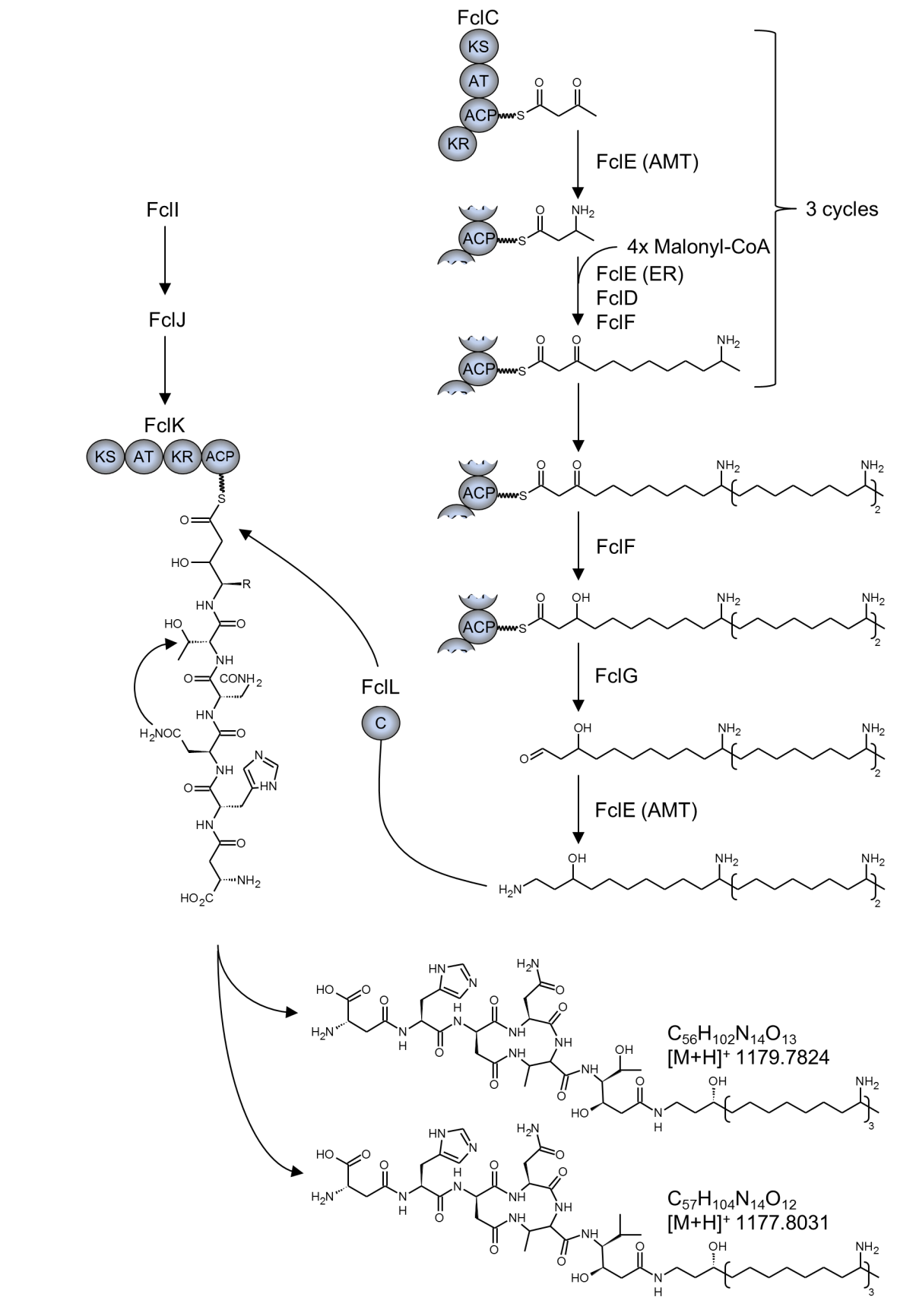


**Figure S1.** Fabclavine biosynthesis in *X. szentirmaii* (modified from [15]). After the generation by the NRPS FclI, FclJ and the PKS FclK, the NRPS-PKS-part stays enzyme-bound until its condensation with the polyamine, catalyzed by FclL [15]. Abbreviations: C: condensation domain, KS: ketosynthase, AT: acyltransferase, ACP: acyl carrier protein, KR: ketoreductase.

**
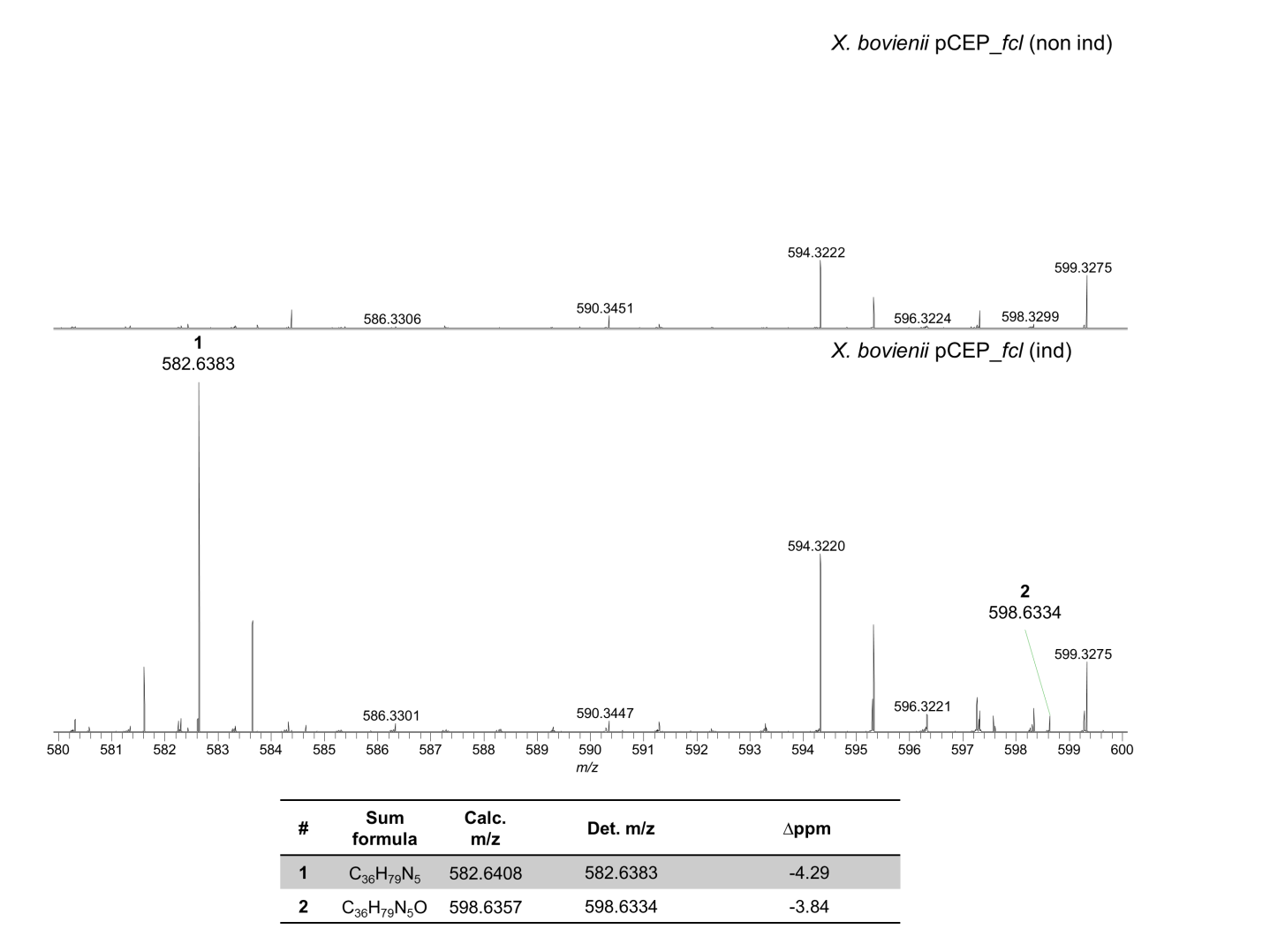
**

**Figure S2.** MALDI-HRMS-spectra of *X. bovienii* pCEP_*fcl* mutant (induced and non-induced) with compounds **1** and **2.** Shown are sum formulas, calculated and detected masses and corresponding ∆ppm. Cultures were grown in LB media for 3 days with constant shaking at 30°C.

**
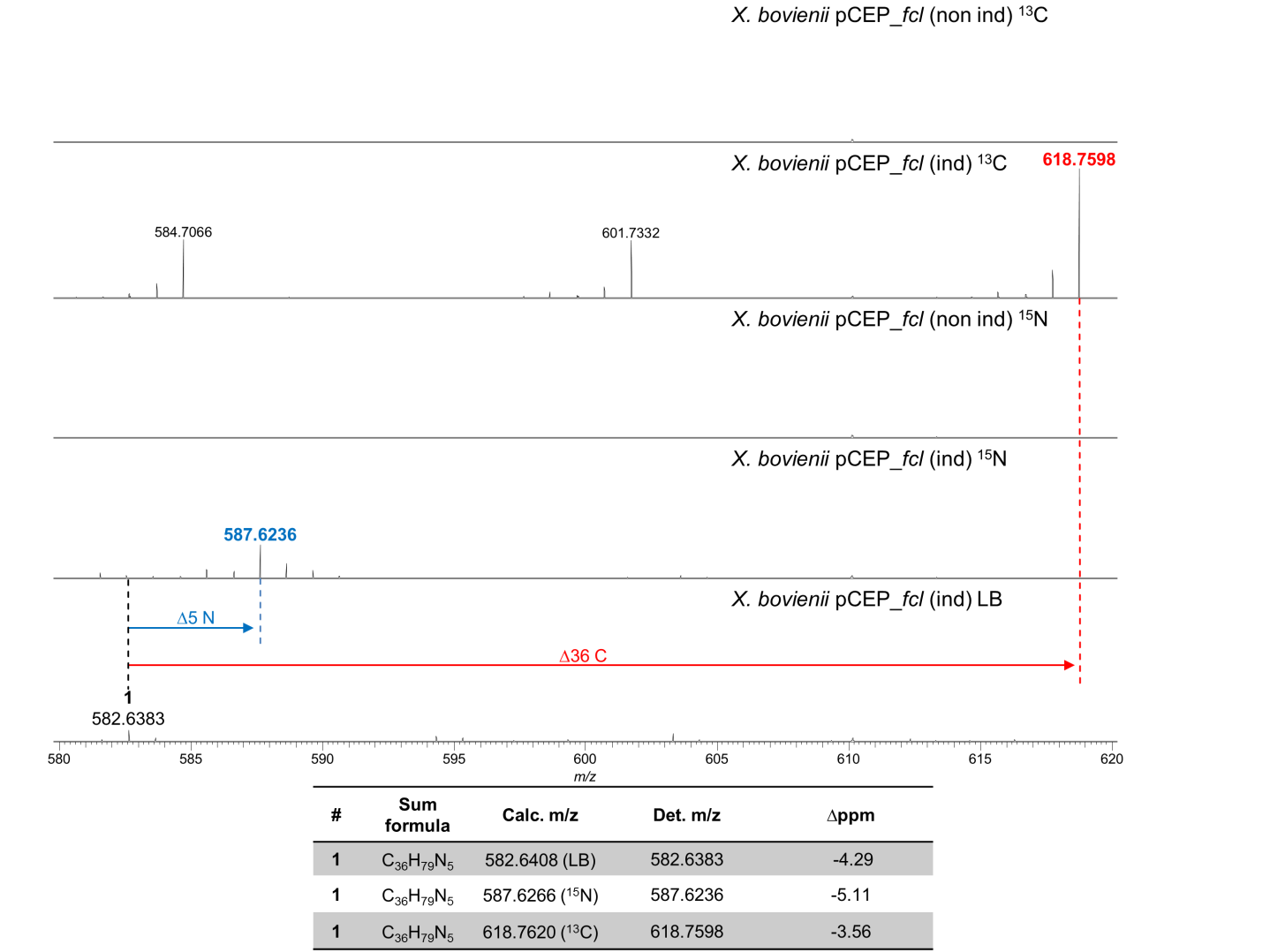
**

**Figure S3.** MALDI-HRMS-spectra of *X. bovienii* pCEP_*fcl* mutant (induced and non-induced) of isotope-labelling experiments compared to *X. bovienii* pCEP_*fcl* (ind) in LB to confirm the sum formula of compound **1**. Cultures in ^15^N- or ^13^C-media were grown for 2 days (LB: 3 days) with constant shaking at 30°C.

**
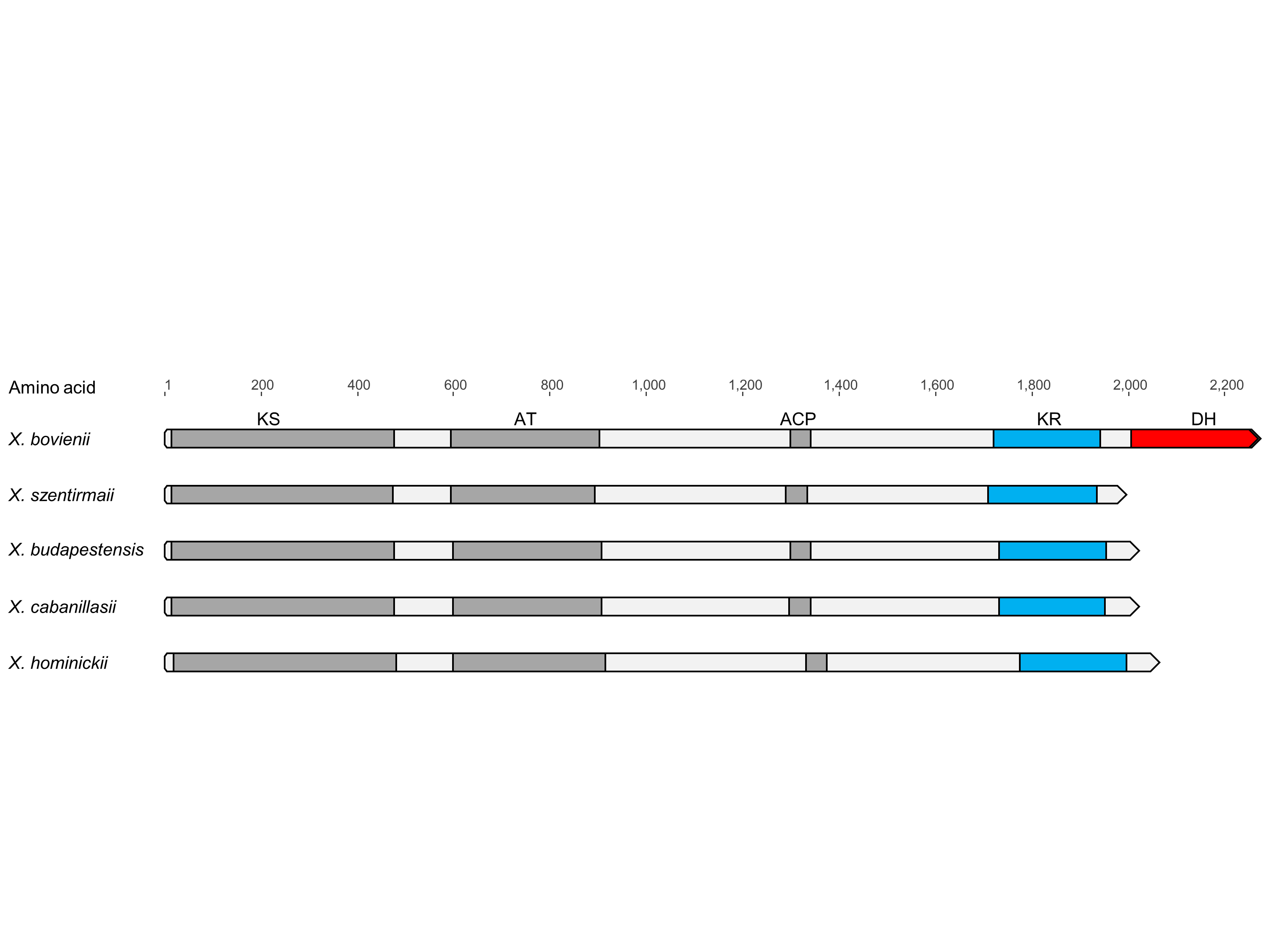
 Figure S4.** Domain comparison of FclC homologues from multiple *Xenorhabdus* strains. KS: ketosynthase, AT: acyltransferase, ACP: acyl carrier protein, KR: ketoreductase, DH: dehydratase

*X. hominickii* **WGPWD**GGMVSDVLKRAYEAQNMVIIPLEEGIQRFVREFRDPRSCQITLGGET**Y**K**A**P**K**KTNISRNNNRYHSPS---

*X. szentirmaii* **WGPWD**GGMVGDVLKRAYQAQNMVIIPLEEGTQRFVHEFGDHRSRQITIGGKT**Y**G**A**V**K**TLLPVK------------

*X. bovienii* **WGPWD**GGMVSDVLKRVYDAQNMAIIPLEEGTQRFVREFRDPGSLQITIGGAN**Y**K**A**E**K**TVKALGIGE**QVDRQLSLD**…

*X. budapestensis* **WGPWD**GGMVSDVLKRAYEAQNMVIIPLDEGIKRFVREFSDPRSRQITIGGKT**Y**M**A**P**K**KTIPNKLRNVINNAAI--

*X. cabanillasii* **WGPWD**GGMVSDVLKRAYEAQNMVIIPLDEGTKRFVREFSDPRSRQITIGGKT**Y**M**A**P**K**KTIPNKLRNVINNAAI--

**Figure S5.** Detailed alignment of FclC homologues to identify the YxAxK-motif. Analyses were performed by the online tool Clustal Omega [Version 1.2.4]. The KR domain is marked in blue, the DH domain in red and the YxAxK-motif in green.


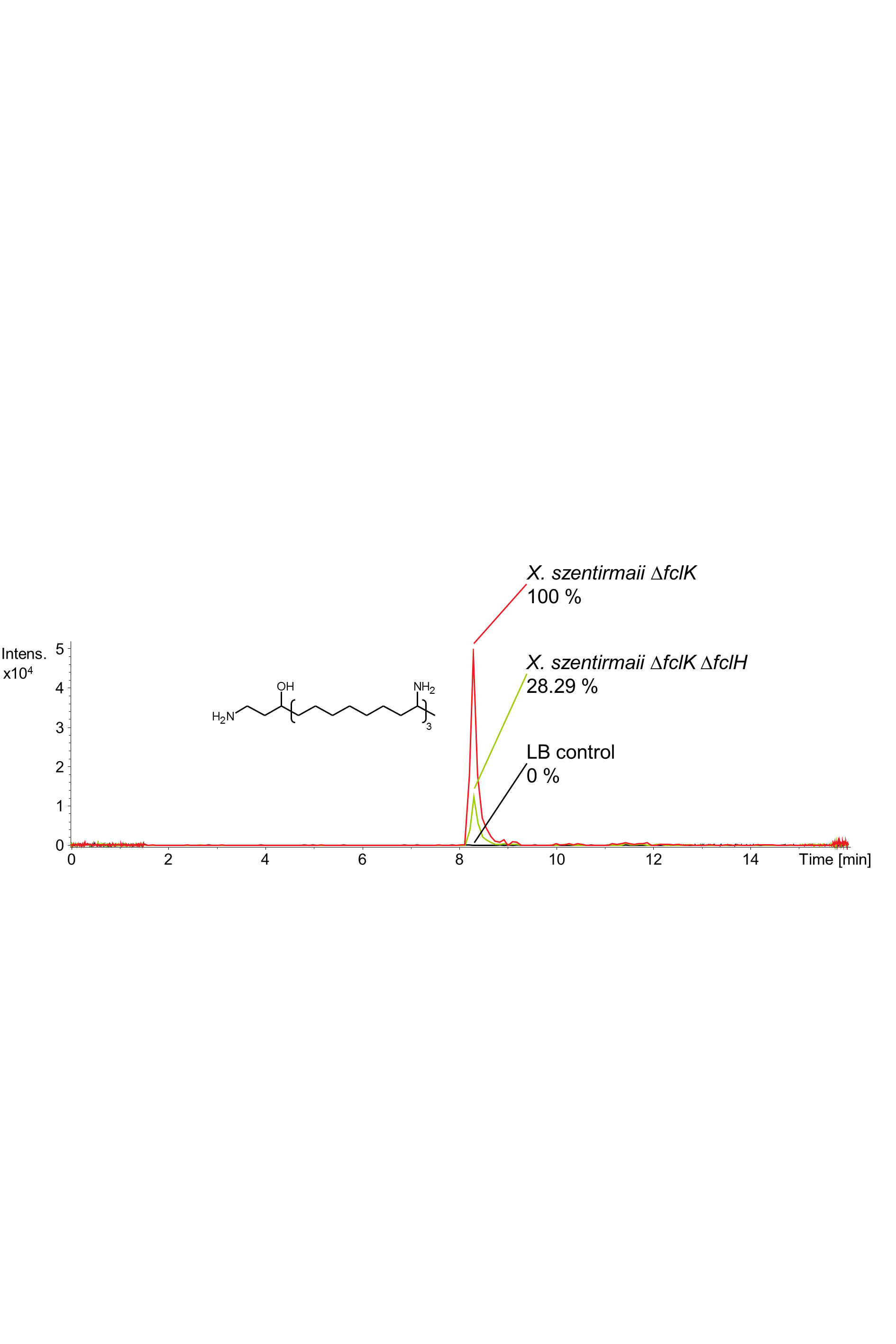


**Figure S6.** Influence of FclH on the production titer of the polyamine in *X. szentirmaii*. Production cultures in LB-media of the strains *X. szentirmaii* ∆*fclK* w/o ∆*fclH* and a LB control were measured in triplicates via HPLC-HRMS. The production titer was relatively quantified by comparing the average of the integrated peak area in the extracted ion chromatogram (EIC) of C_28_H_62_N_4_O [M+H]^2+^.

1 msetyflhdr kvrgdiaivg mashfpdapd lykfweniig kkdsltdvst mlgdeywqke

61 dfydpnpava dktyghragf vppidfdpve fkippaiids istaqlfaly vakqamldag

121 lvgqensrvd rdrigvilgg agngntsfsl asrqqapylr kimvksglse kvandiierm

181 hgmylewned sfpgflgnva cgriasyfdl ggtsymvdaa casslaaika aigelhsgsc

241 davltggvnl ensifsflcf sktpalsksn lsrpfdqsad gmmlgdgvgm lvlkrledae

301 ldgdriyavi ksieassdgr aksifaprle gqakalrray asaglspndi qlveahgtgt

361 asgddtelks lhtvfgeyqv paksvaigsi ksqightrca agaasmmkva lalhhkvlpp

421 tlnvdkptnl lkaenspfyv nsearpwlrs fnsaprraal safgfggtnf hvileeyekh

481 thgryrlnes pwvmlfkghn paellaqcee altrfsgnlp diairqhleq qdidslqpqq

541 arvlfvsqsa eqtvellsia ikqlqqnsth gwehprgiyy qpqgkmldgk ivalfpgqgs

601 qyvnmardia ndypemrqsl etldevsise lghelspvvy pvptfsdder qihqqrltdt

661 anaqpalgai sagyfnilkg mgfvpdfvag hsygevtalw aagvfsdknf hrvslargwa

721 aasasdhrga dtdagamlaa slnsaqraqi lerysgiiia ndnsqqqvvf ggatplihql

781 hdelkkrdvh crilpvsaaf htafiepayq pyrerlagin fqspqcrlfs sataepygns

841 pqairellae qlikpvqfrq tieaiyqegg rlfvevgpkg vlgklvadil kdkehevisv

901 npndkgedrl qfaraqakll avgvklrein qhirpqpmae dkskrltfrm sggfylsqkn

961 karrqralrd gdsaiveqfi aesapvpmap vmplhsqkev nikeehsgke vkptqevhpt

1021 qevvvekven kvekiaenkt isaqfiqqnm ltiiqrrest meqnnqlsen vdvnmlngll

1081 qaqqvmsqlh qqfqanqkdy iqllgmlldk qytlletckd hqnlpsmlss lsqsvqlldk

1141 nlelyhsnhe hyfmaqqslf qsgqvtpmia pthrtsspvv dysalakvtt satiasapvs

1201 tpiptpiptt apavkvdtap vqakpvdpik eiepvrsmmp empvmpsvve eavkpivndt

1261 pvsqptpvvi etkakvldpk vekqfqilsq iteekivnql isivsdrtgy pqdmitpemd

1321 leadlgidsi krleifgamf dafsanvgly neasrnkdle nfevdslsni skmgvffkqm

1381 lddvmndllg nggdeavaeq sppsvetreh entksnvvpd sggatgklqs lgfftstvts

1441 peadsvkkpl aepqlivpsv vqpvlkneiv lneeesfags pvsrfavvkq slpmpdrlqg

1501 vfsspkrwlv vdegtttvdd vvnalrqqgq qvavlmlkqn pavmeseaal daaladieqq

1561 hgtiegviyl qtpkqsvkal aevfneqdyr svettfllak rlqrslnhde qktgyfmvim

1621 rgdgelltsg rehlsivsag ltgltkslni ewknvfcrtv didarvkdsd aakmvveelq

1681 dsrtdmgevg rgvngermtl alteenvlsa afvnqvnagd vlivtggarg itaqcviela

1741 kqsqatfill grtditeplp ewaegkttqd erktaamayl kaqgiqltpv kingmlsrlt

1801 hldeingtlq airqaggrvm ylhcditdlk qvkialtkaq qqlgqitgli hgagnladkr

1861 iekktladlh svfnakvkgl enlcreldft slrhimlfss vsgffgnagq adyslanetl

1921 nkfiylpfkg knsqiirsvn wgpwdggmvs dvlkrvydaq nmaiipleeg tqrfvrefrd

1981 pgslqitigg an**ykaek**tvk algige~~qvdr qlsldknpfl yhhviegksv lpmtaaiawm~~

2041 ~~aricedrfpg ykletitdfk vlkgivfdnt laesycvawv pntfdesgdg rlaldvvits~~

2101 ~~krmkhyqatl ilslqsreaq hevvgidlna evpqtlpfyd gvggegilfh gesfhgvqkv~~

2161 ~~vhvdddnitl kccvmpvqle mqgqfstrlf nlfindiafq lpaiwflqrn emclpnaiek~~

2221 ~~ieqyaelkfg qefyasieik hqmsteiiid iifydeqgyi ysrfhnakft v~~fve

**Figure S7.** Sequence of FclC from *X. bovienii* SS-2004. Marked in red is the YxAxK-motif, crossed out is the removed sequence for pFF1_*fclC*(∆DH)*DEFGH* *X. bovienii*, underlined is the sequence for the *X. bovienii* DH domain-integration into pFF1_*fclC*(+DH)*DEFGH* *X. budapestensis* and pFF1_*fclC*(+DH)*DEFGH X. hominickii*. For the co-expression plasmid pACYC_ara_tacI_DH *X. bovienii* the underlined sequence was used inclusive the three C-terminal residues FVE and an N-terminal M.

**
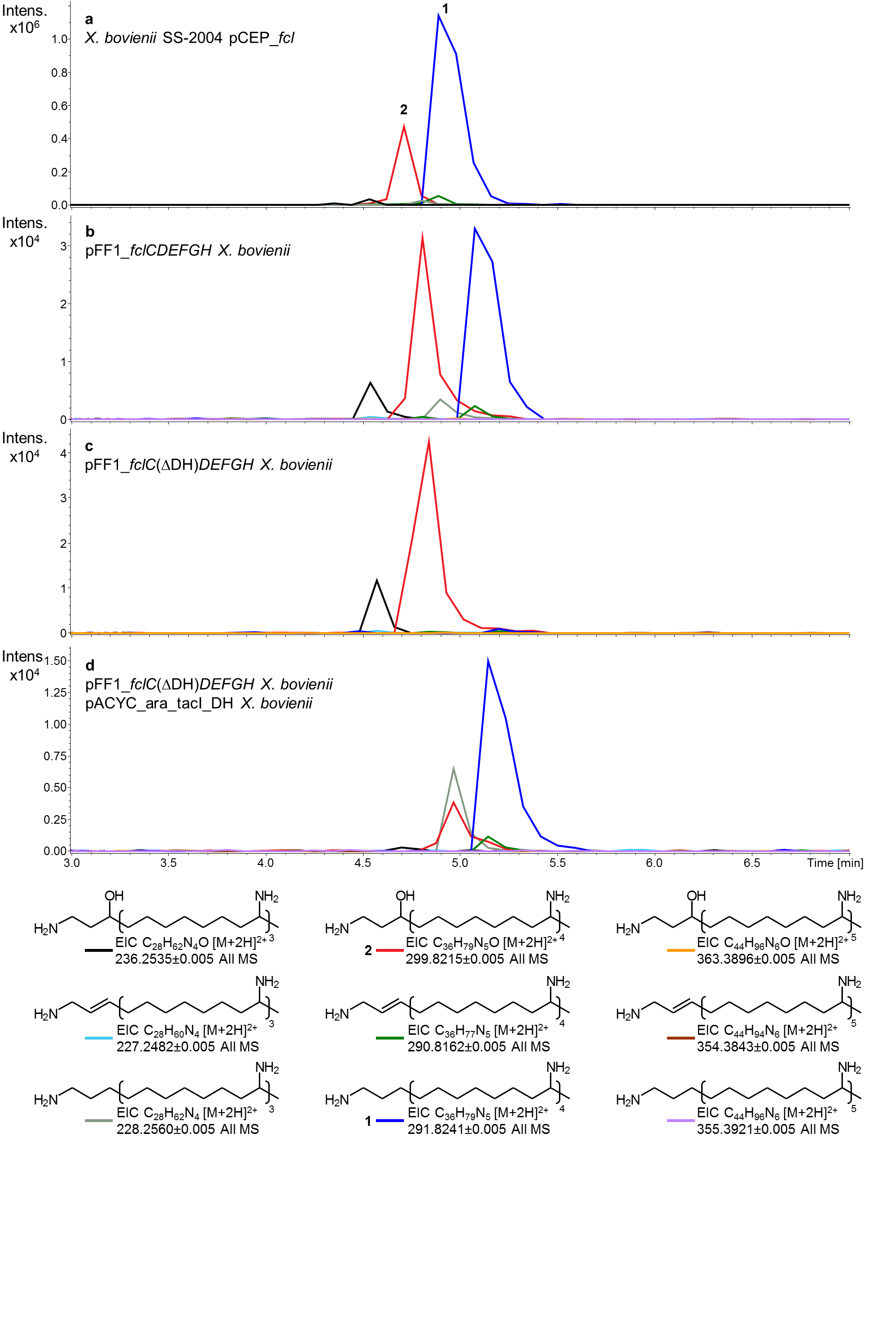
 Figure S8.** HPLC-HRMS analysis of *X. bovienii* pCEP_*fcl* (a) and the heterologous production of *fclCDEFGH* from *X. bovienii* in *E. coli* DH10B::*mtaA* (b-d). Shown are the extracted ion chromatograms (EIC) of the double charged masses. Sample numbers refer to Figure 4.

**
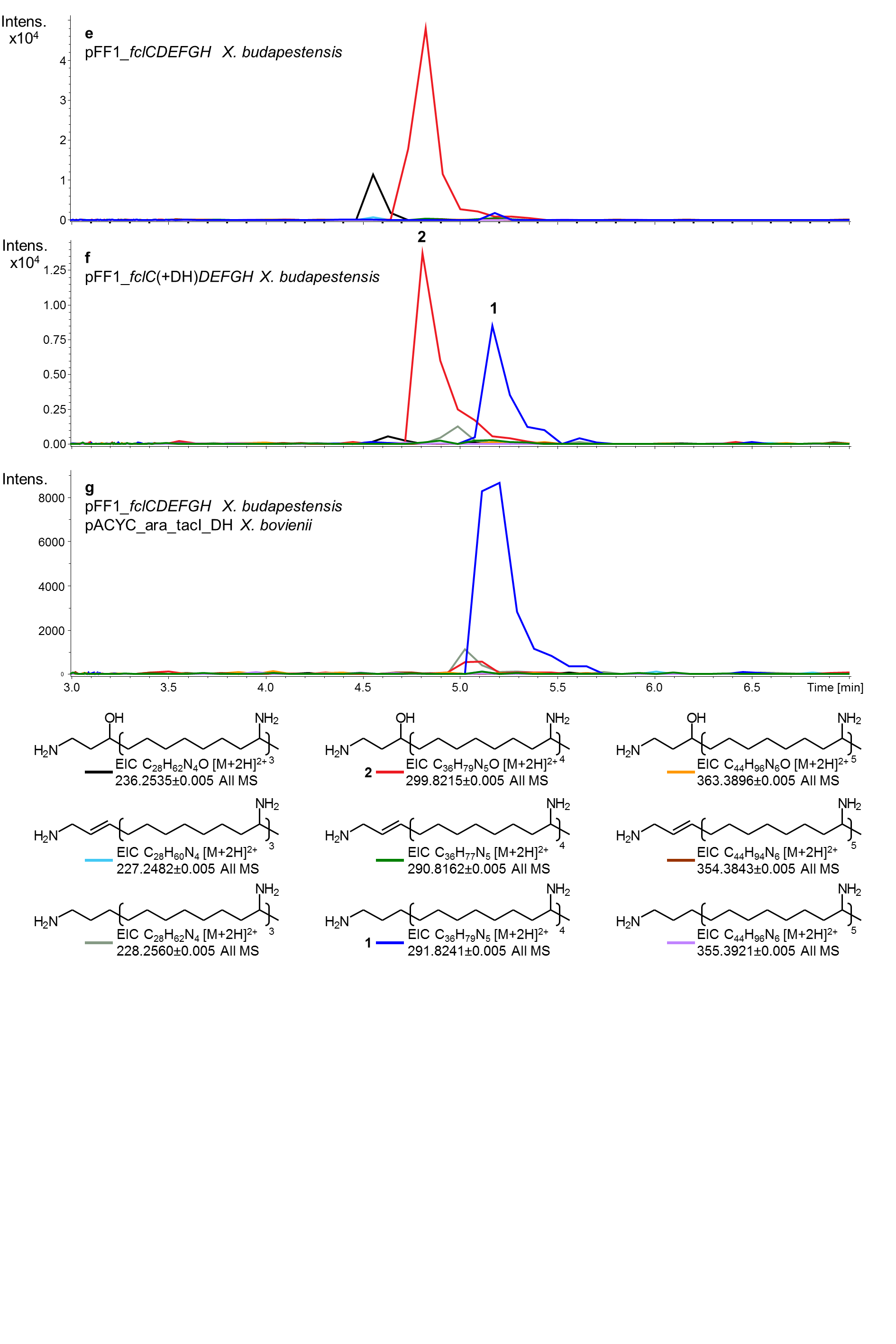
 Figure S9.** HPLC-HRMS analysis of the heterologous production of *fclCDEFGH* from *X. budapestensis* in *E. coli* DH10B::*mtaA*. Shown are the extracted ion chromatograms (EIC) of the double charged masses. The fused PKS-like DH domain from *X. bovienii* into FclC is marked as (+DH). Sample numbers refer to Figure 4.

**
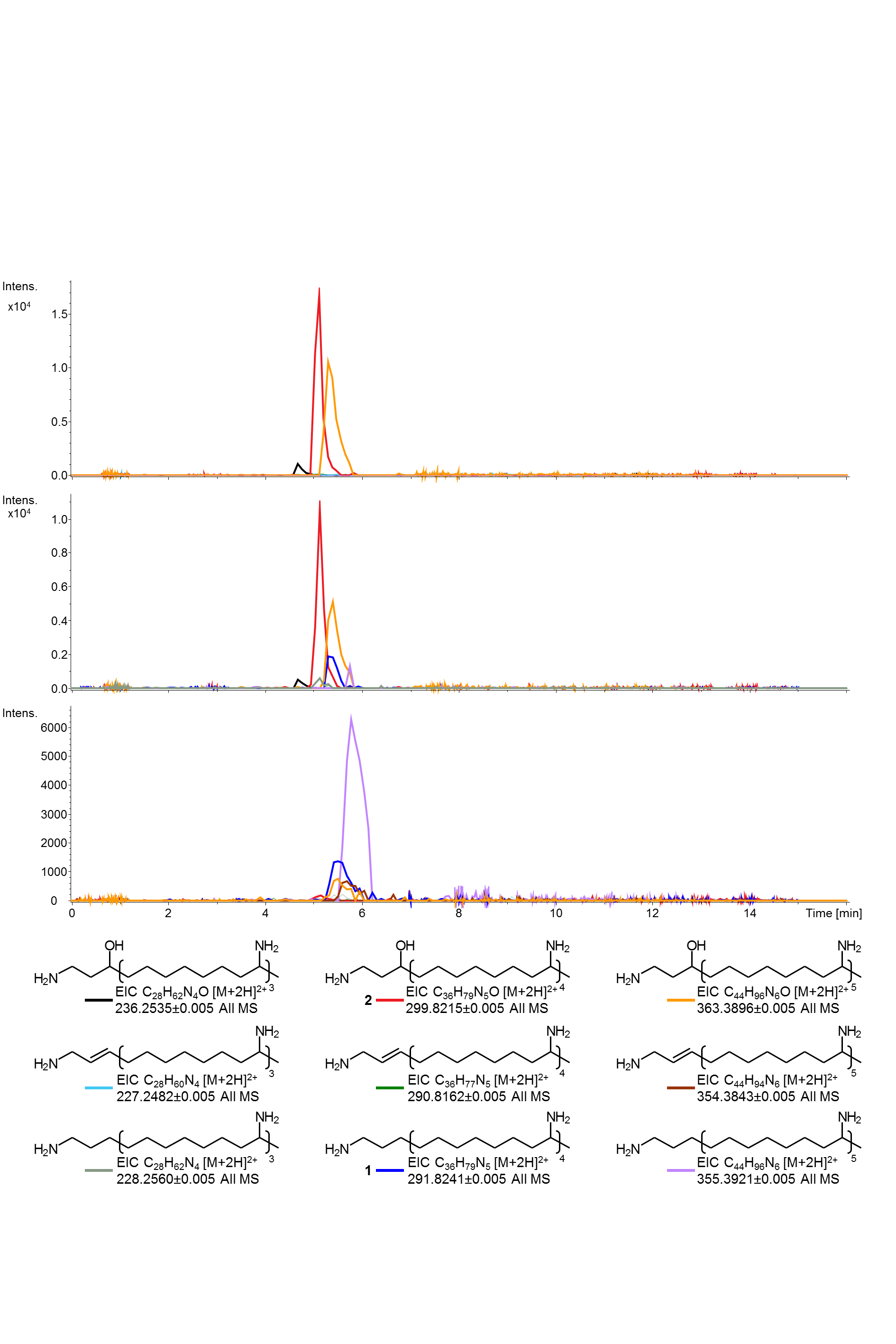
 Figure S10.** HPLC-HRMS analysis of the heterologous production of *fclCDEFGH* from *X. hominickii* in *E. coli* DH10B::*mtaA*. Shown are the extracted ion chromatograms (EIC) of the double charged masses. The fused PKS-like DH domain from *X. bovienii* into FclC is marked as (+DH). Samples were fivefold concentrated.

**
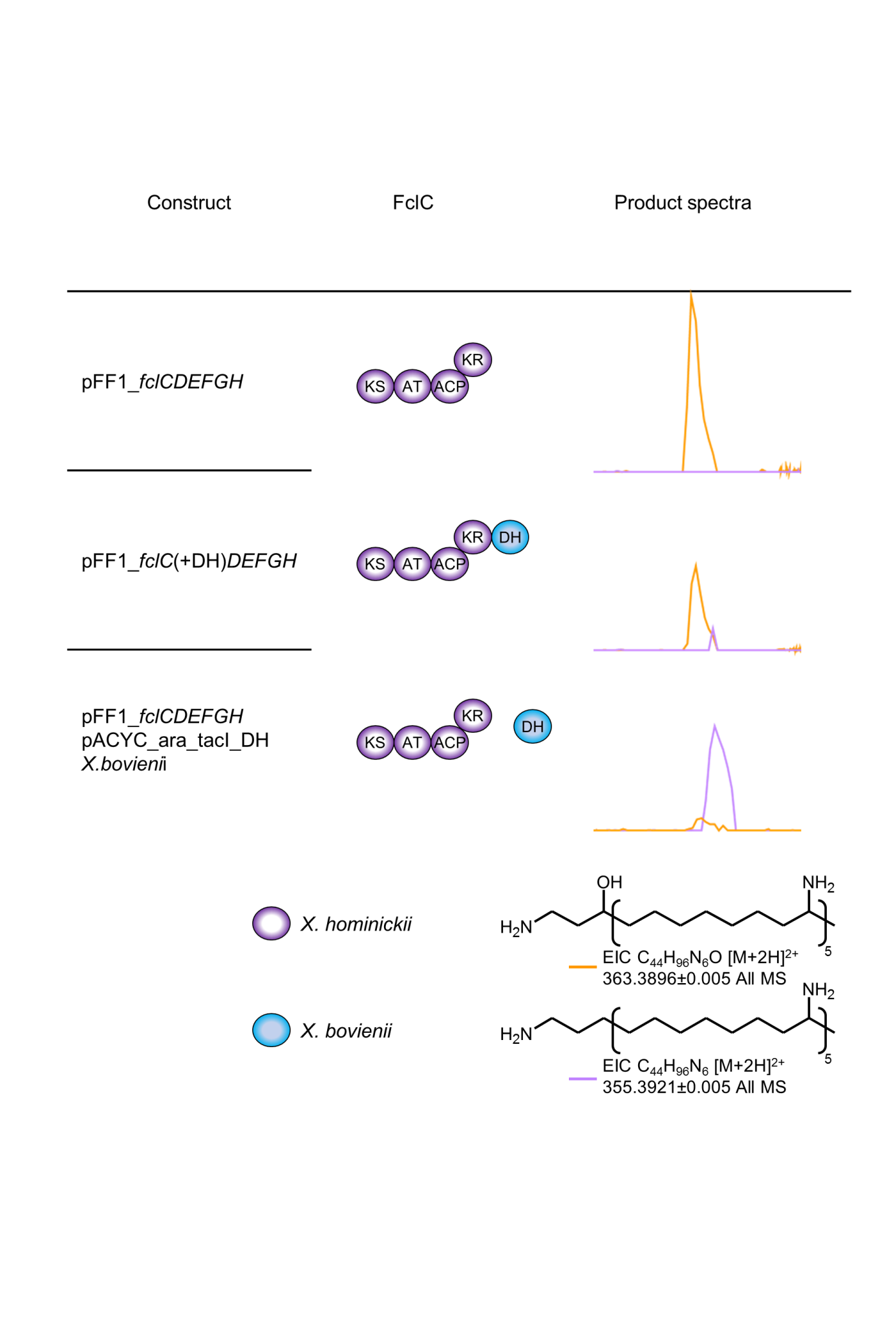
**

**Figure S11.** Manipulation of the polyamine biosynthesis of *X. hominickii*. The genes *fclCDEFGH* from *X. hominickii* were heterologously produced in *E. coli* DH10B::*mtaA.* The fused PKS-like DH domain from *X. bovienii* into FclC is marked as (+DH). Samples were fivefold concentrated.


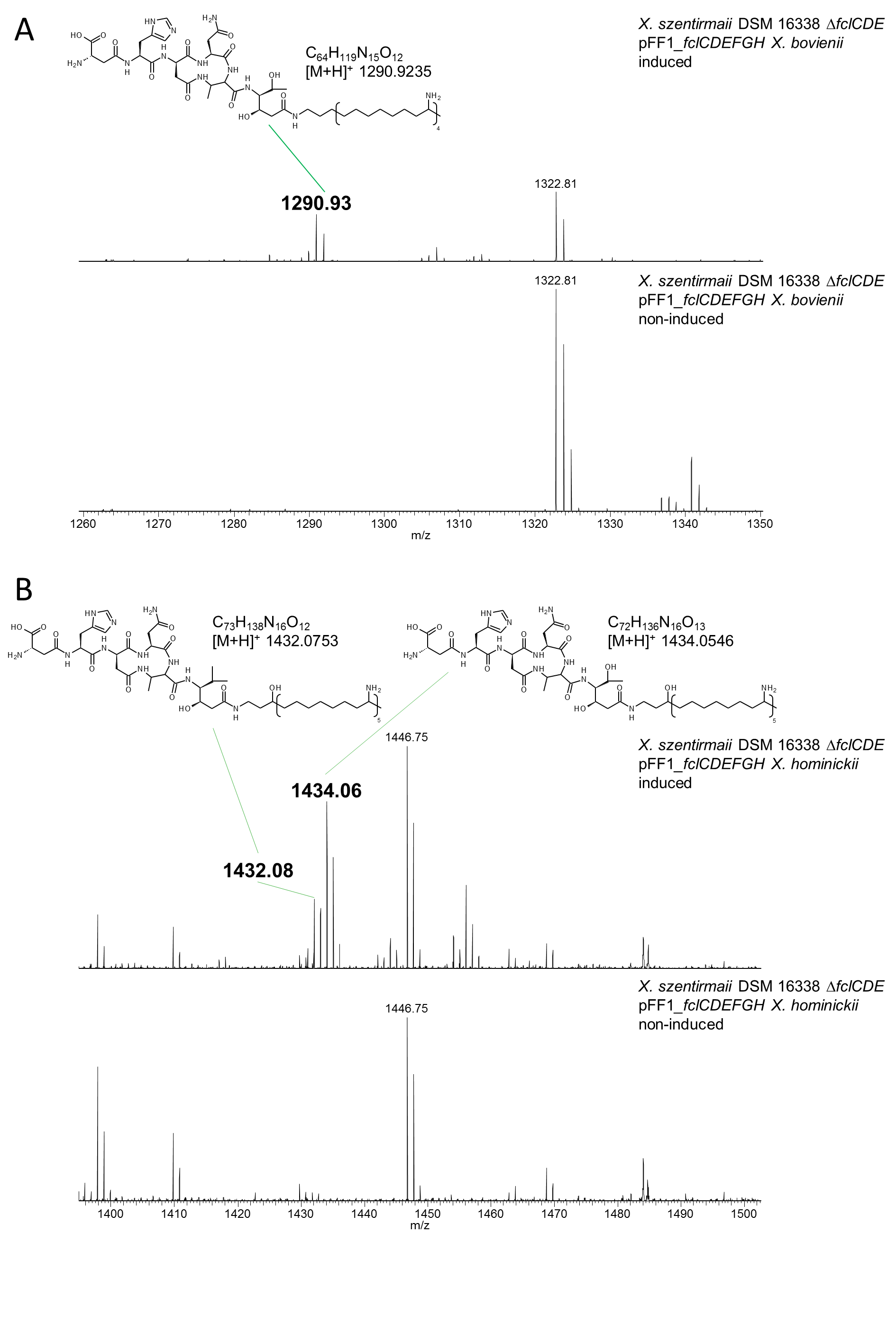


**Figure S12.** Exchange of the polyamine in the *X. szentirmaii* fabclavine biosynthesis. Shown are the MALDI-MS analysis of *X. szentirmaii* ∆*fclCDE* complemented with *fclCDEFGH* of *X. bovienii* (A) or *X. hominickii* (B) and corresponding fabclavine hybrids.

References

1. Bode E, Brachmann AO, Kegler C et al. (2015) Simple “on-demand” production of bioactive natural products. ChemBioChem 16:1115–1119. doi: 10.1002/cbic.201500094

2. Bode E, Heinrich AK, Hirschmann M et al. (2019) Promoter Activation in Δ hfq Mutants as an Efficient Tool for Specialized Metabolite Production Enabling Direct Bioactivity Testing. Angew Chem Int Ed 131:19133–19139. doi: 10.1002/anie.201910563

3. Bozhüyük KAJ, Fleischhacker F, Linck A et al. (2018) De novo design and engineering of non-ribosomal peptide synthetases. Nat Chem 10(3):275-281. doi: 10.1038/nchem.2890

4. Brachmann AO, Joyce SA, Jenke-Kodama H et al. (2007) A type II polyketide synthase is responsible for anthraquinone biosynthesis in *Photorhabdus luminescens*. ChemBioChem 8:1721–1728. doi: 10.1002/cbic.200700300

5. Fu C, Donovan WP, Shikapwashya-Hasser O et al. (2014) Hot Fusion: an efficient method to clone multiple DNA fragments as well as inverted repeats without ligase. PLoS ONE 9:e115318. doi: 10.1371/journal.pone.0115318

6. Gemperlein K, Rachid S, Garcia RO et al. (2014) Polyunsaturated fatty acid biosynthesis in myxobacteria: different PUFA synthases and their product diversity. Chem Sci 5:1733. doi: 10.1039/c3sc53163e

7. Gietz RD, Schiestl RH (2007) Frozen competent yeast cells that can be transformed with high efficiency using the LiAc/SS carrier DNA/PEG method. Nat Protoc 2:1–4. doi: 10.1038/nprot.2007.17

8. Gietz RD, Schiestl RH (2007) High-efficiency yeast transformation using the LiAc/SS carrier DNA/PEG method. Nat Protoc 2:31–34. doi: 10.1038/nprot.2007.13

9. Philippe N, Alcaraz J-P, Coursange E et al. (2004) Improvement of pCVD442, a suicide plasmid for gene allele exchange in bacteria. Plasmid 51:246–255. doi: 10.1016/j.plasmid.2004.02.003

10. Schimming O, Fleischhacker F, Nollmann FI et al. (2014) Yeast homologous recombination cloning leading to the novel peptides ambactin and xenolindicin. ChemBioChem 15:1290–1294. doi: 10.1002/cbic.201402065

11. Simon R, Priefer U, Pühler A (1983) A Broad Host Range Mobilization System for In Vivo Genetic Engineering: Transposon Mutagenesis in Gram Negative Bacteria. Bio/Technology 1:784 EP -. doi: 10.1038/nbt1183-784

12. Thoma S, Schobert M (2009) An improved *Escherichia coli* donor strain for diparental mating. FEMS Microbiol Lett 294:127–132. doi: 10.1111/j.1574-6968.2009.01556.x

13. Tobias NJ, Wolff H, Djahanschiri B et al. (2017) Natural product diversity associated with the nematode symbionts *Photorhabdus* and *Xenorhabdus*. Nat Microbiol 2:1676–1685. doi: 10.1038/s41564-017-0039-9

14. Tobias NJ, Heinrich AK, Eresmann H et al. (2017) *Photorhabdus*-nematode symbiosis is dependent on hfq-mediated regulation of secondary metabolites. Environ Microbiol 19:119–129. doi: 10.1111/1462-2920.13502

15. Wenski SL, Kolbert D, Grammbitter GLC et al. (2019) Fabclavine biosynthesis in *X. szentirmaii*: shortened derivatives and characterization of the thioester reductase FclG and the condensation domain-like protein FclL. J Ind Microbiol Biotechnol. doi: 10.1007/s10295-018-02124-8

16. Wenski SL, Cimen H, Berghaus N et al. (2020) Fabclavine diversity in *Xenorhabdus* bacteria. Beilstein J Org Chem 16:956–965. doi: 10.3762/bjoc.16.84
